# *In silico* characterisation of minor wave genes and LINE-1s transcriptional dynamics at murine zygotic genome activation

**DOI:** 10.3389/fcell.2023.1124266

**Published:** 2023-06-14

**Authors:** Federico Ansaloni, Stefano Gustincich, Remo Sanges

**Affiliations:** ^1^ Area of Neuroscience, Scuola Internazionale Superiore di Studi Avanzati (SISSA), Trieste, Italy; ^2^ Central RNA Laboratory, Istituto Italiano di Tecnologia—IIT, Genova, Italy

**Keywords:** zygotic genome activation, transposable elements (TEs), LINE-1 (L1), MERVL, transcriptional regulation, computational genomics

## Abstract

**Introduction:** In mouse, the zygotic genome activation (ZGA) is coordinated by MERVL elements, a class of LTR retrotransposons. In addition to MERVL, another class of retrotransposons, LINE-1 elements, recently came under the spotlight as key regulators of murine ZGA. In particular, LINE-1 transcripts seem to be required to switch-off the transcriptional program started by MERVL sequences, suggesting an antagonistic interplay between LINE-1 and MERVL pathways.

**Methods:** To better investigate the activities of LINE-1 and MERVL elements at ZGA, we integrated publicly available transcriptomics (RNA-seq), chromatin accessibility (ATAC-seq) and Pol-II binding (Stacc-seq) datasets and characterised the transcriptional and epigenetic dynamics of such elements during murine ZGA.

**Results:** We identified two likely distinct transcriptional activities characterising the murine zygotic genome at ZGA onset. On the one hand, our results confirmed that ZGA minor wave genes are preferentially transcribed from MERVL-rich and gene-dense genomic compartments, such as gene clusters. On the other hand, we identified a set of evolutionary young and likely transcriptionally autonomous LINE-1s located in intergenic and gene-poor regions showing, at the same stage, features such as open chromatin and RNA Pol II binding suggesting them to be, at least, poised for transcription.

**Discussion:** These results suggest that, across evolution, transcription of two different classes of transposable elements, MERVLs and LINE-1s, have likely been confined in genic and intergenic regions respectively in order to maintain and regulate two successive transcriptional programs at ZGA.

## Introduction

Upon fertilisation, the fusion of highly differentiated sperm and oocyte cells generates a totipotent embryo. The newly generated embryo is transcriptionally inactive and, in absence of transcription, its development relies on maternally supplied transcripts and proteins originally deposited into the oocyte cytoplasm (Eckersley-Maslin et al., 2018; Schulz and Harrison, 2019). However, for the embryo to continue its development, zygotic genome activation (ZGA) must occur (Schulz and Harrison, 2019).

ZGA is characterised by a minor and a major transcriptional waves which, in *Mus musculus* (mouse), occur in the early 2-cell and 2-cell stages respectively (Tadros and Lipshitz, 2009; Lee et al., 2014; Jukam et al., 2017). Murine ZGA appears to be activated by the transcription factor (TF) *Dux* (encoded by the *Duxf3* gene) which coordinates a specific transcriptional programme characterised by the expression of, among others, the *Zscan4*, *Prame*, *Eif1a*-like gene family members and of the endogenous retroviruses (ERVs) MERVLs (De Iaco et al., 2017; Hendrickson et al., 2017). MERVL elements, however, are not simply *Dux* targets since they act as potent ZGA activators by themselves. By providing alternative promoter sequences, MERVLs activate the transcription of many ZGA minor wave genes thus enabling a robust and coordinated transcriptional activation of the zygotic genome (Peaston et al., 2004; Macfarlan et al., 2012; Torres-Padilla, 2020).

In addition to MERVLs, long interspersed nuclear element 1 (LINE-1), another class of transposable elements (TEs), play crucial roles during murine ZGA (Jachowicz et al., 2017; Percharde et al., 2018). Evolutionary young LINE-1 transcripts (A, G_*f*_ and T_*f*_ subfamilies) act as chromatin remodellers in the 2-cell stage (Jachowicz et al., 2017). In particular, LINE-1 function appears to be necessary and stage-specific as both the elongation of LINE-1 transcription beyond the 2-cell stage and its transcriptional repression immediately after fertilisation lead to failures in embryonic development (Jachowicz et al., 2017). In addition, recent evidence described how LINE-1 RNAs, together with Nucleolin and Kap1 proteins, are required to repress *Dux* and its 2-cell stage-specific transcriptional program (Percharde et al., 2018).

In summary, MERVL elements activate a ZGA-specific transcriptional program and LINE-1 RNAs are required to switch it off, allowing the embryo to develop beyond this stage. This model leaves several important questions unanswered. How is the transcription of LINE-1 elements activated at ZGA and from which loci? Is this linked to the *Dux*/MERVL transcriptional program or LINE-1s are activated by an independent program?

In this study, we integrated publicly available RNA-seq, ATAC-seq and Stacc–seq datasets to characterise the dynamics regulating the activation of MERVL and LINE-1 elements at murine ZGA at the transcriptional and epigenetic levels. We identified and characterised a set of LINE-1 elements which result likely transcribed at ZGA minor wave onset, according to our analyses of ATAC-seq and Pol-II binding site datasets. We propose that the transcription of these LINE-1s might be regulated by YY1. Altogether, our results show that MERVL elements are transcribed from gene-dense regions while LINE-1 RNAs are expressed from intergenic regions, suggesting the interplay of two, distinct, transcriptional programs co-ordinately regulating murine ZGA.

## Materials and methods

### RNA-seq data collection

The RNA-seq data analysed in this study were retrieved from a previous publication (Wu et al., 2016). The RNA-seq dataset is composed by 5 different samples: MII-oocyte, zygote, early 2-cell (30 h post fertilization [hpf]), 2-cell (39–43 hpf), 4-cell (54–56 hpf) and 8-cell (68–70 hpf) stages. Each stage is represented by two biological replicates. Reads are in paired-end (PE) layout (2 × 126bp). Raw RNA-seq reads were retrieved from the ENA-EBI database (accession number: PRJNA277361), the technical replicates corresponding to the same experiment were merged. Next, the quality of the raw reads was assessed using FastQC (Andrews, 2010). Having detected the presence of both adapters and low-quality reads, the sequencing reads were trimmed using Trimmomatic (v0.38, parameters: ILLUMINACLIP:{adapter.fa}:2:30:10:2:keepBothReads, LEADING:5 TRAILING:5, SLIDINGWINDOW:4:15 MINLEN:30) (Bolger et al., 2014).

### Gene expression quantification

Trimmed RNA-seq reads were mapped to the murine genome (mm10 version—gencode vM22 version) using STAR (v2.6.0c) (Dobin et al., 2013). Default parameters were used except for the number of multimapping reads that was set to 80 (--outFilterMultimapNmax 80). Reads were counted by using htseq-count (v0.11.2, parameters: --stranded no -m union --nonunique all) (Anders et al., 2015).

### TE expression quantification—locus specific

TE locus specific expression levels were calculated using SQuIRE (Yang et al., 2019). First, the reference genome and the annotation datasets referring to the murine mm10 genome version were downloaded and prepared for the subsequent analyses using the SQuIRE Fetch and Clean modules, then the trimmed reads were mapped to the reference genome using the Map module and finally read counts were estimated using the Count module (strandedness = “0”). Only TEs annotated as DNA, LINE, SINE, LTR and RC were selected for subsequent analyses. TEs annotated as “MERVL-int”, “MERVL_2A-int”, “MT2A″, “MT2B″, “MT2B1”, “MT2B2”, “MT2C_Mm” and “MT2_Mm”, were classified as MERVL.

### TE expression quantification—consensus level

Expression levels of the TE consensus were calculated using TEspeX (Ansaloni et al., 2019; 2022). Here is reported a brief description of the TEspeX workflow. The reference transcriptome was built merging the RepBase TE sequences (Bao et al., 2015) and the Ensembl transcript sequences containing all the coding and non-coding annotated transcripts (Zerbino et al., 2018). Reads were then mapped on the reference transcriptome using STAR (v2.6.0c) (Dobin et al., 2013) assigning primary alignment flag to all the alignments with the best score. All alignments flagged as primary (−F 0 × 100 parameter) were then selected using samtools (v1.3.1) (Li et al., 2009). To avoid counting reads mapping to TE fragments embedded in coding and/or long non-coding transcripts, reads mapping with best-scoring alignments to any Ensembl transcript were discarded using Python scripts and Picard FilterSamReads (v2.18.4) (http://broadinstitute.github.io/Picard). Selected reads mapping exclusively on TEs and in the proper orientation were finally counted in each sample.

### Differentially expressed gene analyses

Differentially expressed genes (DEG) between the early 2-cell and the zygote stages were identified using edgeR (Robinson et al., 2010). Normalisation of raw read counts was applied using the TMM method whereas the common, trended and tagwise dispersions were estimated by maximizing the negative binomial likelihood (default). Next, DEG were tested performing a quasi-likelihood F-tests (glmQLFit and glmQLTest). Genes were considered as differentially expressed (DE) if showing FDR < 0.05 and log2FC < −1 or > 1. The same workflow was used for all the DE gene analyses and also to identify the DE TE (single loci). DE TE consensus sequences were identified by using the same workflow previously described with the exception that the library size of each sample was calculated providing the total number of reads mapped on the TEspeX transcriptome (coding, non-coding and TE consensus sequences) instead of using the default values.

### GO enrichment analysis

GO enrichment analysis was performed using topGO (Alexa and Rahnenfuhrer, 2019). GO enrichment analysis was conducted on the GO terms associated to the early 2-cell/zygote upregulated genes, using as background the GO terms associated to the whole set of coding and non-coding annotated genes. First, the statistical significance of the enrichments was tested with the Fisher’s Exact Test (algorithm = “weight”). Then, GO terms associated to less than 15 significant genes were discarded prior to FDR calculation (Benjamini and Hochberg). Significant threshold was imposed to FDR < 0.05.

### Upregulated gene TSS enrichment analysis

Transcription start sites (TSS) of the upregulated genes were defined as the first nucleotide at the 5’ of each given gene. To identify TEs enriched nearby the TSS of the early 2-cell vs. zygote upregulated genes, the TSS of each gene was elongated both at the 5’ and 3’ by 100 nucleotides. The genomic coordinates of the elongated TSS were then overlapped with the genomic coordinates of all the murine TEs using bedtools intersect (v.2.27.0) (Quinlan and Hall, 2010) and its python wrapper pybedtools (Dale et al., 2011). Finally, the number of genes overlapping each TE subfamily was counted. The same analysis was repeated selecting an equal number of randomly selected genes for 1,000 times. Z-scores and *p*-values were then calculated and corrected using the FDR Benjamini and Hochberg correction. FDR significant threshold was set to 0.05.

### Identification of gene clusters in the murine genome and overlap with upregulated genes

Gene clusters in the murine genome were identified using ClusterScan (parameters: -n 5 -d 500,000 –singletons) (Volpe et al., 2018). Next, the number of minor/major wave genes overlapping at least one gene cluster calculated by overlapping the gene and cluster coordinates by bedtools intersect (parameter: -f 0.5). The same analysis was repeated by shuffling 1,000 times the coordinates of the gene clusters using bedtools shuffle. Z-scores, *p*-values and FDR were calculated and filtered as already described.

### RNA-seq from oocyte treated with alpha-amanitin

Raw RNA-seq data was retrieved from two previous studies (Wang et al., 2019; Asami et al., 2023). In the first dataset (Wang et al., 2019), denuded GV oocytes were treated with alpha-amanitin and RNA was isolated and sequenced at the 2-cell stage (4 biological replicates, SE layout, 75 bp). In the second dataset (Asami et al., 2023), embryos were generated by sperm-oocyte mixing for 1 h and then incubated in KSOM containing α-amanitin (100 μg/mL) for a further 10 h, when bipronuclear embryo samples (1-cell stage) were collected (4 biological replicates, PE layout, 2 × 50 bp). RNA-seq reads were treated as previously described and TE expression was calculated at the consensus level using TEspeX (Ansaloni et al., 2022) as previously described.

### ATAC-seq data collection

ATAC-seq data were retrieved from a previous publication (Wu et al., 2016) (accession number: PRJNA277362). The dataset is composed by a total of 5 stages (2 biological replicates, PE layout, 2 × 101bp). The stages were matched with the ones of the RNA-seq except for MII-oocyte ad zygote stages that were missing in the ATAC-seq data as it is technically challenging to extract enough chromatin to perform an ATAC-seq at these stages. To overcome this issue, the authors of the paper treated early zygotes (PN3—pronucleus phase 3) in CZB medium supplemented with alpha-amanitin for about 14 h (Wu et al., 2016). Alpha-amanitin is an inhibitor of the RNA-pol-II, thus alpha-amanitin treated zygotes are transcriptionally inactive stages and likely represent a pre-ZGA sample. Having detected the presence of both adapters and low-quality reads, the ATAC-seq reads were trimmed using Trimmomatic as previously described.

### Identification of ATAC-seq peaks

Trimmed ATAC-seq reads were mapped to the murine genome (mm10 version—gencode vM22 version) using bowtie2 (v2.3.5.1) (Langmead and Salzberg, 2012). Default parameters were used when mapping single-ended reads whereas paired-ended reads were mapped avoiding the selection of both discordant pairs and singleton reads (--no-mixed, --no-discordant parameters). If a sequencing read multimaps to *n* different loci, bowtie2 defines the best alignment (based on mismatches, gaps and other mapping parameters) among these *n* and reports only this to the output file. Next, the Genrich tool was used to perform the ATAC peak calling analysis merging the biological replicates (v0.6, parameters: -j -d 250 -r -a 0 -q 0.05 -e chrM and genomic scaffolds) (https://github.com/jsh58/genrich). Only ATAC peaks longer than 10 nucleotides were retained for further analyses. The same analysis was repeated by using bwa (v 0.7.15-r1140), bowtie1 (v 1.2.3; --chunkmbs 200 --sam --best) and bowtie2 to rule out possible biases introduced by the random assignment of non-uniquely mapping reads.

### ATAC peaks annotation

ATAC peaks were annotated respective to the transcript genic features using the R/Bioconductor package ChIPseeker (v1.24.0) (Yu et al., 2015). The TxDB object was generated from the gtf annotation file used for previous analyses (gencode vM22 version, primary assembly) using the R/Bioconductor package GenomicFeatures (v1.40.1) (Lawrence et al., 2013). ATAC peaks were next annotated using the ChIPseeker annotatePeak function defining a promoter region of +/− 3 kb (tssRegion = c(-3,000, 3,000)). In case of ATAC peaks overlapping more than one genomic feature, priorities were assigned following the ChIPseeker default parameters (promoter, 5’ UTR, 3’ UTR, exon, intron, downstream, intergenic). In order to define how many of the intergenic ATAC peaks were associated to TEs, the genomic coordinates of the ATAC-seq peaks were overlapped with the genomic coordinates of the TEs annotated in the mm10 genome (downloaded from repeatmasker). The overlap was performed by using bedtools intersect (v2.27.0, parameters: -f 0.5, -wao) (Quinlan and Hall, 2010). Since it might happen that one single ATAC-seq peak overlaps more than one annotated TEs, the output file of the bedtools intersect was furtherly parsed in order to reduce this redundancy. Namely, in case of ATAC-seq peaks overlapping more than one annotated TEs, the longest intersection was selected.

### LINE-1 monomer annotation

Genomic coordinates of the LINE-1 monomers were retrieved from a previous publication (Zhou and Smith, 2019). Monomers were then associated to the closest starting coordinate of the annotated LINE-1 elements by using bedtools closest (parameters: -s, -D ref). A given monomer was associated to the closest LINE-1 starting coordinate if: 1) the two features were on the same strand and 2) the monomer overlapped the LINE-1 starting coordinate or was located no more than 100 nucleotides downstream to it. Of note, while performing this analysis we discovered that in many cases two portions of the same LINE-1 element were annotated as distinct LINE-1s by repeatmasker. For instance, in our TE annotation file, two different LINE-1s were annotate at coordinates chr1:3037726–3043047 and chr1:3043047–3044040 and they represented the coordinates of the L1Md_A consensus sequence 279–5,586 and 5,587–6,580, respectively. We believe that these two elements actually belong to a unique LINE-1 element as the two elements are contingent and also represent contingent portions of the respective consensus. To fill this gap, we used custom code to join all the contingent LINE-1s in the murine genome if: 1) the given LINE-1s belonged to the same LINE-1 subfamily (e.g., L1 Md_A), 2) the distance between the two elements on the genome was less than 2 nucleotides and 3) the distance between the represented portions on the LINE-1 consensus sequence was less than 5 nucleotides.

### LINE-1 expression

To define whether the young LINE-1s overlapping early2-cell intergenic ATAC peaks were expressed during the developmental time-course, the expression levels of the LINE-1s of interest were compared to the expression levels of randomly selected young LINE-1s. To this end, the normalised expression levels of the LINE-1s of interest were retrieved from the previous TE single loci expression quantification analysis (see “TE expression quantification—locus specific” section). Expression of contingent LINE-1s whose coordinates were joined (see “LINE-1 monomer annotation”) was summed. The same analysis was then repeated 1,000 times on an equal number of randomly selected young LINE-1s regardless of their ATAC status in the early2-cell stage. This analysis was performed separately for young LINE-1s with (n = 2,116) and without (n = 1,298) a monomer.

### Stacc-seq data collection

Stacc-seq data were retrieved from a previous publication (accession number: PRJNA558961) (Liu et al., 2020). In total, the dataset was composed by: MII-oocyte (13 hpf), PN2 1-cell (23 hpf), PN3 1-cell (26 hpf), PN5 1-cell (30 hpf), early 2-cell (36 hpf), late 2-cell (48 hpf) and 8-cell (71 hpf). In order to match these stages with the ones previously analysed in the RNA-seq and ATAC-seq dataset, we retrieved 5 stages: 1) MII-oocyte, corresponding to MII-oocyte of the RNA-seq dataset; 2) PN3 1-cell embryos, corresponding to the zygote stage of the RNA-seq dataset; 3) PN5 1-cell embryos, corresponding to the early 2-cell stage of the RNA-seq and ATAC-seq dataset (they were both collected at 30 hpf, but in the Stacc-seq dataset is called “PN5 1-cell”, whereas in the RNA-seq and ATAC-seq it is called “early 2-cell”); 4) late 2-cell, corresponding to the 2-cell stage of the RNA-seq and ATAC-seq datasets and v) 8-cell stage, corresponding to the 8-cell stage of the RNA-seq and ATAC-seq datasets. Each stage is represented by two biological replicates. Reads are in PE layout (2 × 150bp). Having detected the presence of both adapters and low-quality reads, the Stacc-seq reads were trimmed using Trimmomatic as previously described.

### Identification of Pol-II binding sites

Mapping to the reference genome, peaks identification and bigwigs files generation were done as previously described for the ATAC-seq data with the exception that no threshold was set on the minimum length of the Pol-II binding sites and that only binding sites scoring an area under the curve (AUC) higher than 500 were selected for further analyses.

### Enrichment of Pol-II binding sites on LINE-1 monomers

Genomic coordinates of the monomers of the LINE-1s of interest were elongated by 100 nucleotides at both far ends and intersected (bedtools intersect) with the genomic coordinates of the Pol-II binding sites identified in each of the analysed stages. The same analysis was repeated by shuffling 1,000 times the coordinates of the Pol-II binding sites by using bedtools shuffle. Z-scores, *p*-values and FDR were calculated as previously described. The same analysis was also repeated intersecting the end-point coordinate of the same LINE-1 elements. Upset plots were generated by using the ComplexUpset R library (Krassowski et al., 2022), ggplot2 extension of UpSetR (Conway et al., 2017), based on the “UpSet” technique firstly conceived by (Lex et al., 2014).

### Motif enrichment analysis

Motif enrichment analysis was performed by using the findMotifs tool of the Homer package (Heinz et al., 2010). Motifs were searched in the monomer sequences of the LINE-1s of interest (evolutionary young LINE-1 overlapping early 2-cell intergenic ATAC peaks and carrying a monomer) using as background the sequences of evolutionary young LINE-1 overlapping early 2-cell intergenic ATAC peaks and not carrying a monomer. To validate the results of this analysis, the same analysis was repeated using the *runAme()* function of the memes R package, which is a wrapper of the AME tool of MEME Suite (McLeay and Bailey, 2010). Motif enrichment analysis on the LINE-1 sequences overlapping early 2-cell intergenic ATAC-seq peaks was instead performed using as background the nucleotide sequence of the portion of the LINE-1s of interest not overlapping any ATAC-peaks.

### Metagene plot

**FIGURE 1:**
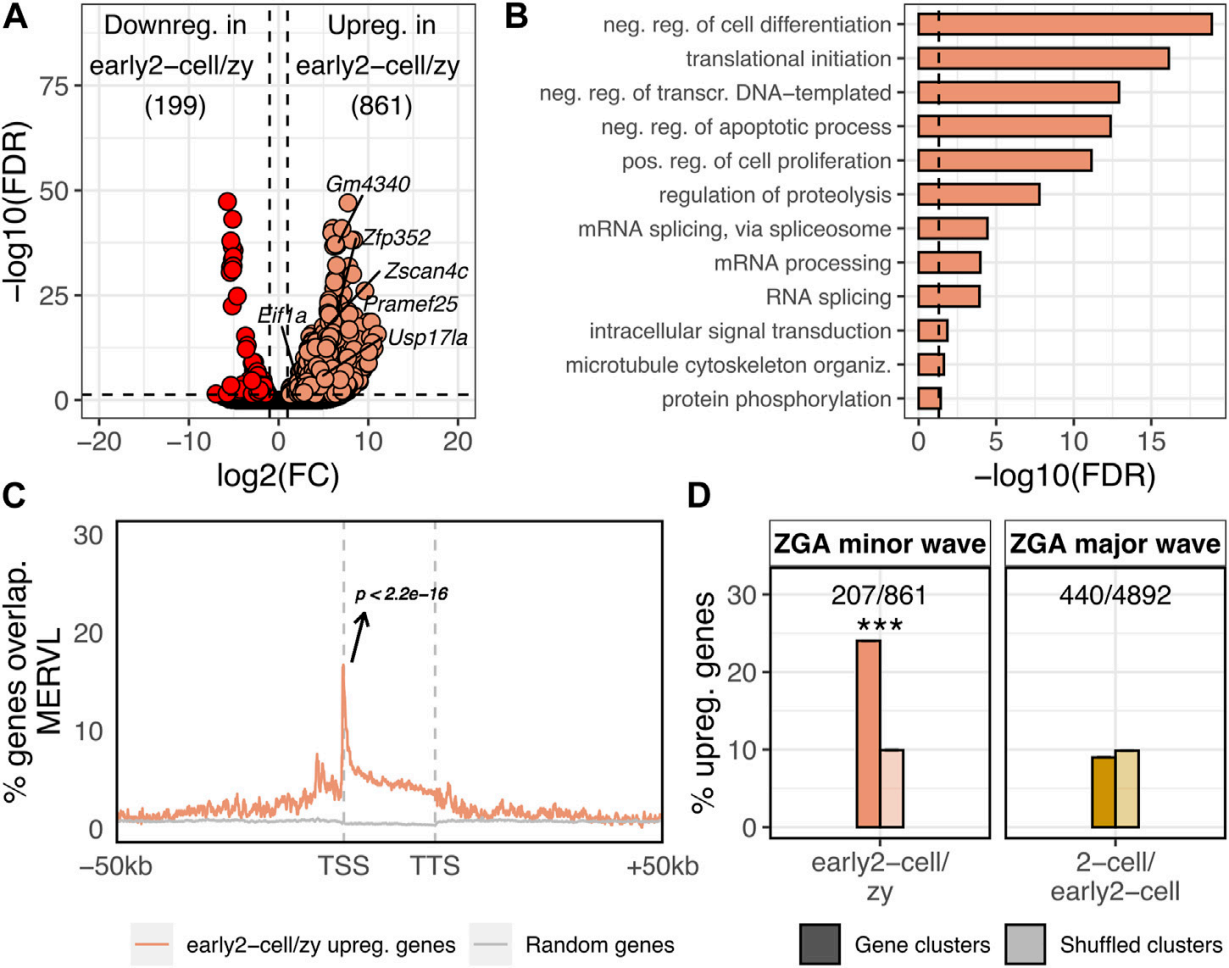
ZGA minor wave genes are enriched in MERVL sequences and located within gene clusters. **(A)** Volcano plot showing DE genes between early 2-cell and zygote stages. Up and downregulated genes in early 2-cell vs. zygote stages are reported. log_2_FC is reported on x-axis, whereas -log_10_ of the FDR on the y-axis. Significantly downregulated genes are coloured in red, the upregulated in light orange (FDR<0.05 and log_2_FC > 1 or < -1). Gene names are reported for 2C-specific genes. **(B)** GO terms enriched in the set of genes upregulated in early 2-cell vs. zygote. Only GO terms relative to biological process (BP) are reported. Significant threshold set to FDR<0.05. **(C)** Metagene showing the MERVL enrichment nearby the 861 genes upregulated in early 2-cell _*vs*_ zygote. MERVL enrichment is reported on y-axis as the fraction of the 861 genes overlapping MERVL sequences at a given position. The same is reported for randomly selected genes (grey, 1,000 randomisations, mean is reported) (*p* < 2.2e-16, z-statistics). **(D)** Percentage of upregulated genes in early 2-cell vs. zygote (ZGA minor wave) and 2-cell vs. early 2-cell (ZGA major wave) overlapping gene clusters (darker colours). The same analysis was repeated by shuffling (i.e., randomly permuting) 1,000 times the coordinates of the gene clusters (shuffled clusters, lighter colours). In the shuffled bars mean ± sem is reported, however, due to the small variability among the 1,000 randomisations they are not visually appreciable. (Shuffled values are 9.94 ± 0.70 and 9.88 ± 0.031, respectively. ***p = 2.9e-10 and *p* = 0.36, respectively, z-statistics).

The metagene plots represented in Figures 1C, 3D were generated using custom scripts developed in python3. Briefly, the genomic features of interest were subdivided in a given number of bins. Each bin of each genomic feature of interest was then overlapped with the second genomic feature of interest (e.g., MERVL coordinates or ATAC-seq peaks). When at least 50% of the nucleotides of the bin overlapped the investigated element, 1 was assigned to the bin otherwise 0. In the plots, on the y-axis is represented the mean number of genomic features of interest overlapping a given element (e.g., MERVL coordinates or ATAC-seq peaks). This number was multiplied by 100, thus representing the percentage of genomic features of interest covered by the investigated element. The random signal depicted in Figure 1C was obtained applying the same workflow to randomly selected genes (1,000 randomisations). Metagene in Figure 4D was generated by using the computeMatrix scale-regions tool of the deeptools package (Ramírez et al., 2016). The random signal depicted in Figure 4D was obtained applying the same workflow to shuffled (bedtools shuffle) LINE-1 coordinates (1,000 randomisations). Since the genomic tracks at these developmental stages are quite noisy, the bedGraph/bigwig genomic regions falling outside significantly identified peaks (see Genrich analysis) were assigned a value of 0.

### Bigwig track generation

Bigwigs track generated in this study for both ATAC-seq and Stacc-seq (Pol-II binding) data are deposited on Zenodo at: https://zenodo.org/record/7807839#.ZC_N3y8RrEo. The bigwig tracks were generated by using the bamCoverage tool of the deepTools package (v3.5.0, parameters: --binSize 10 --centerReads --ignoreDuplicates --normalizeUsing RPKM --extendReads 250) (Ramírez et al., 2016). In addition, since at these developmental stages chromatin has a very dynamic conformation, bigwig tracks can be very noisy (Wu et al., 2016) and the signal can be of hard interpretation. To overcome this issue, we also generated bigwig tracks by including only reads overlapping ATAC-seq or Stacc-seq significant peaks previously identified by Genrich. To this end, bamCoverage “—blackListFileName” parameter was provided with a bed file containing all the regions of the mm10 genome not overlapping any significant ATAC-seq/Stacc-seq peak elongated at both ends by 1 kb.

### Statistical analysis

All the statistical analyses performed externally to previously reported software (edgeR, topGO) were conducted either in R (v3.6.2) (R Core Team, 2018) or in python (v3.7.6) (Rossum and Drake, 2001) taking advantage of the numpy (Harris et al., 2020) and scipy (SciPy 1.0 Contributors et al., 2020) libraries. All the plots were generated in R, using either generic R plotting functions or the ggplot2 library (Wickham, 2016). All the *p*-values were FDR corrected (Benjamini–Hochberg). *p < 0.05, **p < 0.01, ***p < 0.001.

### Naming of different groups of LINE-1s

In this article, “old L1” refers to all the LINE-1s not belonging to A, G_*f*_ and T_*f*_, “young L1” refers to the LINE-1s belonging to A, G_*f*_ and T_*f*_ and “LINE-1” refers to all the LINE-1, with no distinctions among evolutionary ages.

## Results

### Genes transcriptionally activated during ZGA minor wave are enriched in MERVL elements and in gene clusters

To identify the genes transcriptionally activated during the minor wave of the murine ZGA, we performed a differentially expressed (DE) gene analysis between the early 2-cell (ZGA minor wave stage) and the zygote (transcriptionally inactive) stages. A total of 1,060 genes resulted differentially expressed between the two conditions with 861 genes being upregulated in early 2-cell compared to zygote (Figure 1A; Supplementary Table S1, FDR<0.05, log2FC > 1 or < -1). These 861 upregulated genes represented the set of genes transcriptionally activated upon the ZGA minor wave. Consistent with this observation, the 861 upregulated genes were significantly enriched in the “2C genes” gene set previously defined by Macfarlan and others (Macfarlan et al., 2012) (Supplementary Table S1, *p* < 2.2e-16) with several of the well-known *Dux* targets belonging to the set of upregulated genes (e.g., *Zscan4*, *Prame*, *Gm4340, Dub1* [*Usp17la*]*, Zfp352*, *Eif1a*-like gene family members). Gene ontology enrichment analysis revealed that, in agreement with the analysed biological context, the upregulated gene functions were mainly related to GO terms such as transcription regulation, translation initiation, proteolysis and cell death/proliferation (Figure 1B). Confirming previous evidence (Macfarlan et al., 2012), the 861 ZGA minor wave genes were significantly associated with MERVL (*p* < 2.2e-16), with the transcription start site (TSS) of 147 of these genes (17%) located in the proximity (<100 nt) of annotated MERVLs, while this is expected by chance for less than 1% of genes in the murine genome (Figure 1C; Supplementary Tables S1, S2). Although the percentage of ZGA minor wave genes associated with MERVL sequences is not predominant, ZGA minor wave gene enrichments in MERVL elements resulted both highly significant and specific as these genes resulted enriched in no other TE sequences (Supplementary Table S2; Supplementary Figure S1).

Next, we wondered whether other genomic features, besides MERVL enrichment nearby the TSS, characterised the ZGA minor wave genes. In particular, starting from previous evidence showing how high gene/promoter density facilitates the transcription initiation at zebrafish ZGA (Hadzhiev et al., 2023), we tested whether the murine ZGA minor wave genes were located in gene-dense compartments. First, gene clusters were defined in the murine genome as genomic compartments characterised by at least 5 consecutive genes associated to the same functional domain (see Methods, Supplementary Table S3). Then, the number of ZGA minor wave genes located within the clusters was calculated. ZGA minor wave genes resulted significantly enriched in gene clusters with respect to the rest of the transcriptome, with 207 of the 861 ZGA minor wave genes (24%) being located within a cluster, while this was expected by chance for only ∼83 genes (10%) (Figure 1D; Supplementary Table S1, *p* = 2.9e-10). To define whether this was a feature specific of the minor wave genes or it was characteristic also of other gene sets, we identified the ZGA major wave genes (upregulated genes in 2-cell vs*.* early 2-cell stage) and overlapped their genomic coordinates with the ones of gene clusters. Of note, cluster enrichment appeared to be ZGA minor wave-specific, as ZGA major wave genes did not display the same enrichment (Figure 1D, *p* = 0.36).

### MERVL elements are the most frequently activated TEs at ZGA minor wave

**FIGURE 2:**
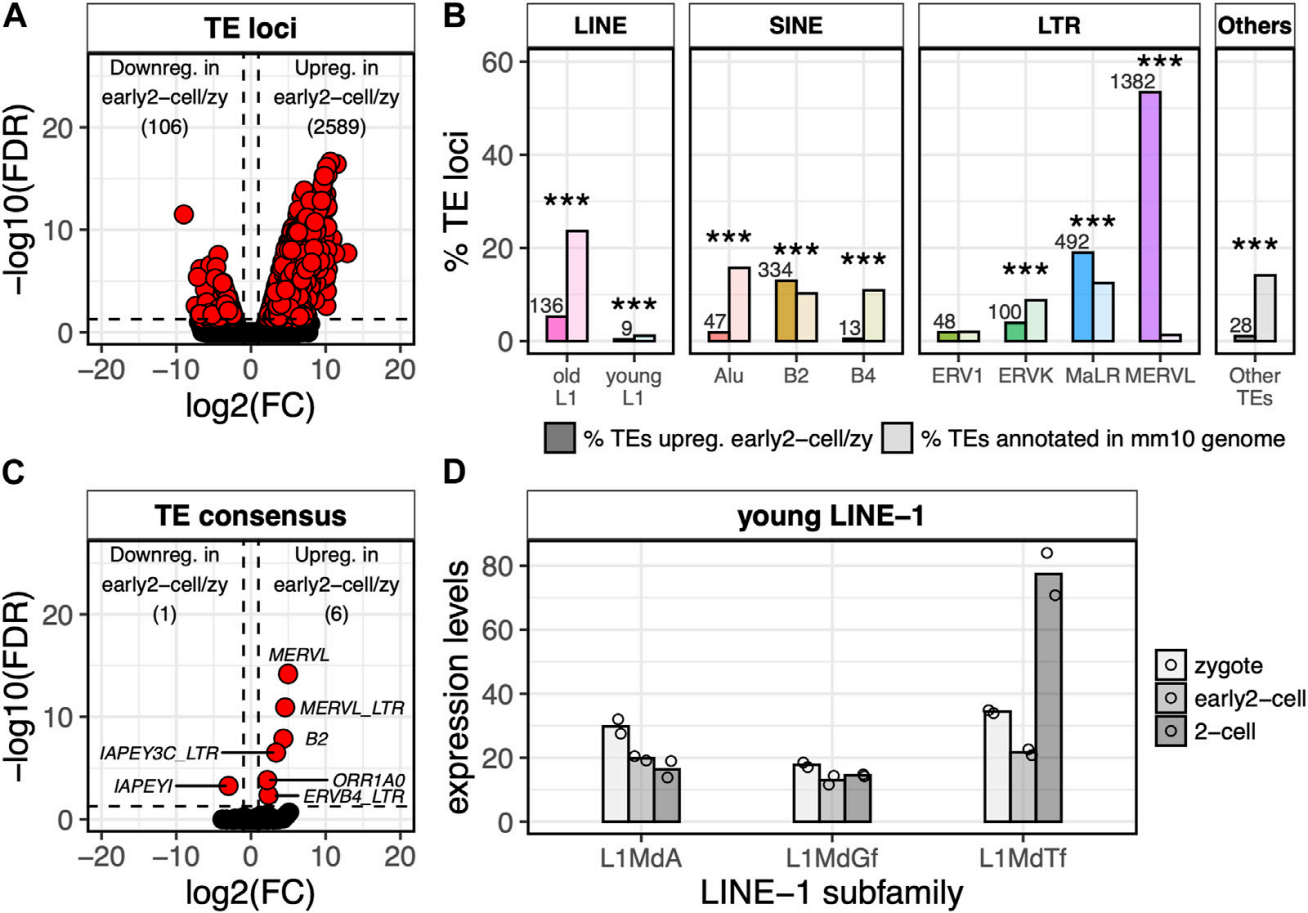
MERVL, but not LINE-1, transcription is detected at ZGA minor wave onset. **(A)** Volcano plot showing DE TE loci between early 2-cell and zygote stages. Up- and downregulated TE loci in early 2-cell vs. zygote stages are reported. log_2_FC is reported on x-axis, whereas -log_10_ of the FDR on the y-axis. Significantly downregulated genes are reported in the left part of the plot, the upregulated in the right one (FDR<0.05 and log_2_FC > 1 or < -1). **(B)** Fraction of upregulated TE loci in early 2-cell vs. zygote stages for each TE subfamily compared to the genome occupancy of each TE subfamily. Darker bars indicate the number of TEs upregulated for each TE subfamily divided by the total number of TEs upregulated, multiplied by 100. Lighter bars indicate the number of TEs annotated in the mm10 genome or each TE subfamily divided by the total number of TEs in the mm10 genome, multiplied by 100. The number of upregulated TEs for each TE subfamily is reported above each bar. “young LINE-1s” are considered LINE-1 elements belonging to A, G_*f*_ and T_*f*_ subfamilies. “old LINE-1” instead refers to all the other LINE-1 subfamilies. (***p < 0.001, two-proportions z-test). Number of TEs annotated in the mm10 genome (lighter bars) are as follow: old L1 863,564 (23.6%), young L1 41,612 (1.1%), Alu 574,557 (15.7%), B2 372,923 (10.2%), B4 397,726 (10.9%), ERV1 71,980 (2.0%), ERVK 319317 (8.7%), MaLR 454918 (12.4%), MERVL 46168 (1.3%), Other TEs 515,652 (14.1%). **(C)** Volcano plot showing DE TE consensus between early 2-cell and zygote stages. Significantly downregulated genes are reported in the left part of the plot, the upregulated in the right one (FDR<0.05 and log_2_FC > 1 or < -1). TE subfamily names are reported for all the DE TEs. **(D)** Normalised expression levels (TMM) of the young LINE-1 element consensus sequences (subfamilies A, G_*f*_, T_*f*_) as calculated by TEspeX in the zygote (light grey), early 2-cell (grey) and 2-cell (dark grey) stages. Bars indicate mean of expression across different LINE-1 consensus sequences belonging to the same subfamily (e.g., L1MdA_I, II, III, IV, etc.) and among the two biological replicates (individual points).

Having identified the genes transcriptionally activated at ZGA minor wave, we sought to define the TEs that were transcriptionally activated at the same stage. To this end, we identified the TE loci differentially expressed between the early 2-cell and the zygote stages. A total of 2,695 TEs resulted DE between the two conditions and, of these, 2,589 were upregulated and only 106 were downregulated in early 2-cell compared to zygote (Figure 2A; Supplementary Table S4; Supplementary Figure S2A, FDR<0.05, log2FC > 1 or < -1). As expected, MERVL elements were the TEs most frequently upregulated at this stage with 53% (1,382/2,589) of the upregulated TE loci being annotated as MERVL, while they represent only 1% of the total TEs in the murine genome (Figure 2B; Supplementary Figure S2A, *p* < 2.2e-16). In addition, in line with previous evidence (Taylor and Pikó, 1987; Bachvarova, 1988; Peaston et al., 2004), the upregulated TEs resulted enriched also in MaLR (observed 19%, expected 12%, *p* < 2.2e-16) and B2 elements (observed 13%, expected 10%, *p* < 2.2e-16) (Figure 2B). The remaining upregulated TEs were mostly old LINE-1s (not belonging to A, G_*f*_ and T_*f*_ subfamilies), ERVs (ERVK and ERV1) and SINE elements (Alu and B4) (Figure 2B; Supplementary Figure S2A). However, these TE subfamilies are amongst the most common in the murine genome and none of these resulted significantly over-represented in the set of upregulated TEs (Figure 2B). The majority of the old LINE-1s resulting significantly upregulated in early 2-cell compared to zygote were evolutionary ancient LINE-1s belonging to the Lx subfamily (Supplementary Figure S2B). Since it is unlikely that these LINE-1s have maintained an autonomous internal promoter, it is reasonable to believe that they are transcribed as part of nearby/overlapping transcripts. This suggestion is supported by the evidence that they result located closer to annotated transcripts than shuffled LINE-1s obtained by randomly permuting these LINE-1 coordinates among the genome (*t*-test *p* = 1.84E-08) (Supplementary Figure S2C).

Of note, almost no evolutionary young LINE-1 elements (A, G_*f*_ and T_*f*_ subfamilies) resulted upregulated in early 2-cell compared to zygote (Figure 2B; Supplementary Figure S2A). The same results were confirmed at the TE consensus level by using TEspeX, a tool which discriminates between TE expression and passive transcription of exonised TE fragments embedded in annotated transcripts (Ansaloni et al., 2019; 2022) (Figure 2C; Supplementary Table S5; Supplementary Figure S3). This result might appear contrasting previous observations showing that mature LINE-1 transcripts are required at the 2-cell stage for proper embryo development (Jachowicz et al., 2017). Here, the small number of biological replicates (i.e., 2) might have compromised the statistical power of the differential expression analysis. However, this apparent discrepancy can be solved when keeping into account that Jachowicz and others collected 2-cell embryos at 46 hpf (Jachowicz et al., 2017), whereas in the dataset herein analysed early 2-cell embryos were collected at 30 hpf (Wu et al., 2016). Taken together, these observations indicate that the LINE-1 transcripts needed at 46 hpf are likely not yet transcribed or, at least, mature at 30 hpf.

Furthermore, it is also worth noticing how LINE-1 RNAs are part of the set of transcripts deposited in the oocyte cytoplasm ahead of fertilisation and consequently part of the zygote cytoplasm (Peaston et al., 2004; Malki et al., 2014). We confirmed this by measuring the amount of young LINE-1 transcripts in the transcriptionally inactive zygote (Figure 2D) and by analysing embryos where transcription was blocked prior to ZGA with alpha-amanitin (Supplementary Figure S4). From this analysis LINE-1 transcripts resulted detectable in the cytoplasm of the embryos even when zygotic transcription was blocked. LINE-1 transcripts were therefore already present in the zygote prior to ZGA.

The presence of LINE-1 transcripts prior to ZGA makes it impossible to define, by only analysing transcriptomic data, whether LINE-1 transcription is actually started between the zygote and the early 2-cell stage. To overcome these limitations, we decided to integrate ATAC-seq data to investigate the chromatin accessibility of the early embryo genome at ZGA onset especially at LINE-1 loci. This approach is based on the hypothesis that zygotically transcribed mRNAs should require specific chromatin conformations in the zygote facilitating their transcription, while maternal transcripts should not.

### More than 25% of the total open chromatin domains at ZGA minor wave onset reside in LINE-1 elements

**FIGURE 3:**
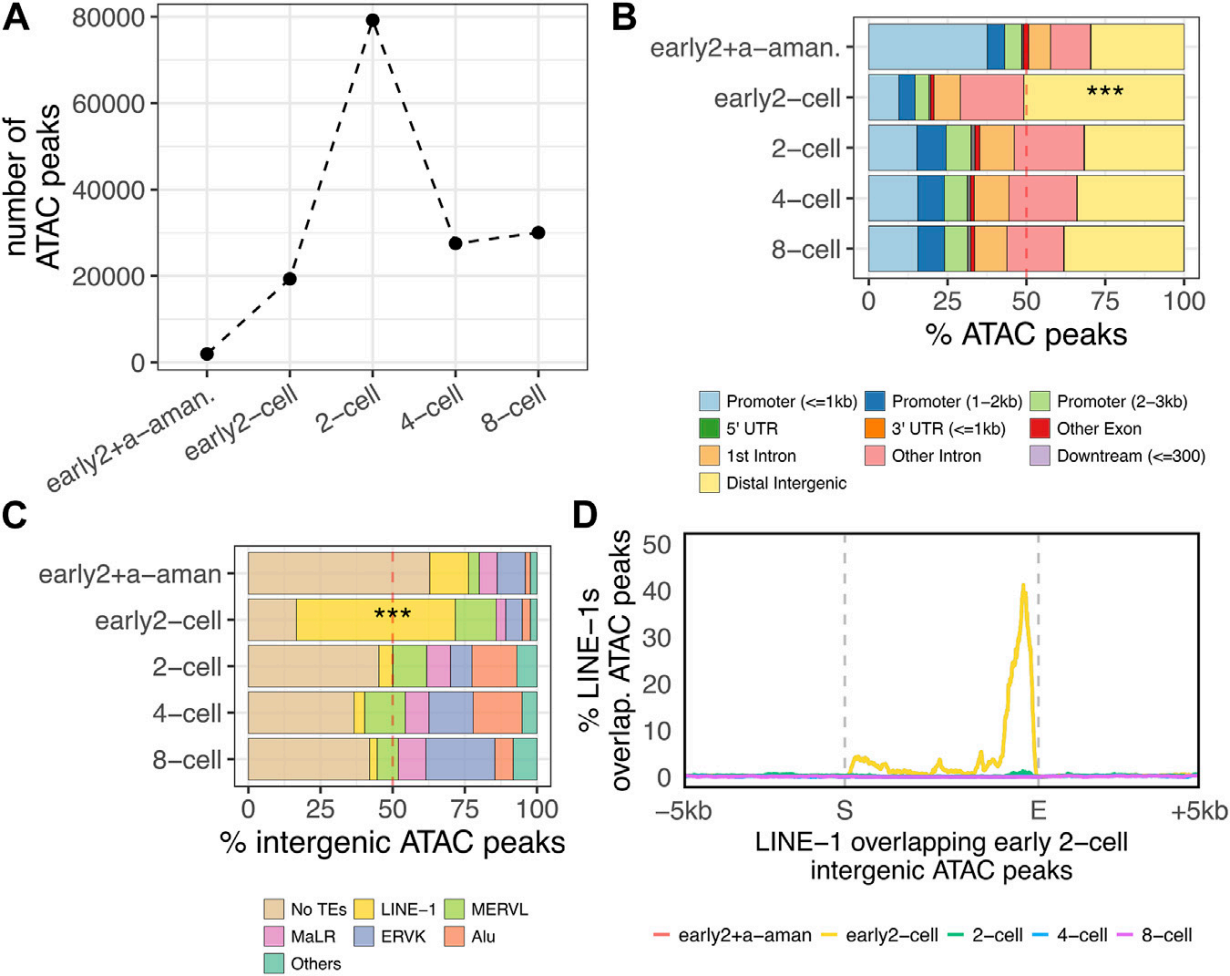
Accessible chromatin at the ZGA onset. **(A)** Number of open chromatin domains during preimplantation embryo development. Number of significant (*p* < 0.05) open chromatin domains (ATAC-seq peaks) are reported for each of the analysed stages. Pre-ZGA sample is represented by “early2+a-aman.”, which represents an early 2-cell embryo treated with alpha-amanitin, an inhibitor of Pol-II. ZGA minor wave sample is the early 2-cell, whereas the major ZGA wave occurs in the 2-cell stage. **(B)** Distribution of ATAC-seq peaks respective to genomic features. The distribution of the ATAC-seq peaks respective to transcript genomic features is reported for each of the analyse stages as percentage of the total peaks identified in each analysed stage (***p < 0.001, two-proportions z-test). **(C)** Fraction of intergenic ATAC-peaks overlapping annotated TEs in each analysed stage. Only LINE-1, ERVL, MaLR, ERVK and Alu subfamilies are reported. All the other TE subfamilies are classified as “Others” (***p < 0.001, two-proportions z-test). “LINE-1” here refers to all the LINE-1s annotated in the mm10 genome with no distinctions between old, young, full-length non-full-length. **(D)** Metagene plot showing the localisation of the ATAC-seq peaks on the genomic loci of the LINE-1 elements overlapping early 2-cell intergenic ATAC peaks identified in Figure 3C (n = 3,731). ATAC-seq peak enrichment on LINE-1s of interest is reported on y-axis as percentage of LINE-1s covered by ATAC-seq peak in each position (+/- 5 kb) of the LINE-1s in analysis.

By taking advantage of the ATAC-seq dataset from a previous publication (Wu et al., 2016), we identified the open chromatin domains during mouse preimplantation embryo development (see Methods) (Supplementary Table S6). Our results showed that the number of ATAC-seq peaks increased by 10-fold upon ZGA minor wave (between the early 2-cell + alpha-amanitin and the early 2-cell samples), further increasing in correspondence of the ZGA major wave onset (2-cell stage) and then decreasing in 4- and 8-cell stages (Figure 3A). Importantly, at ZGA minor wave onset (early 2-cell), only 50% of the peaks overlapped genic features, with the remaining portion (9,553 peaks) localised in intergenic regions (Figure 3B, *p* < 2.2e-16). This is in stark contrast with all the other stages where 70% of the peaks resulted annotated in genic regions. A further investigation of the localisation of these 9,553 early 2-cell intergenic peaks showed that more than 50% of them were in correspondence of LINE-1 elements (5,683 peaks overlapping 3,731 LINE-1s) (Figure 3C). This resulted in a strong enrichment of intergenic open chromatin domains within LINE-1 elements at ZGA minor wave onset (Figure 3C, *p* < 2.2e-16). Additionally, such enrichment resulted stage-specific with no other analysed embryonic stage displaying a similar pattern. The results were confirmed either by repeating the analysis of the same dataset with the same tool (bowtie2) or by mapping the reads to the reference genome using other two tools (bwa and bowtie1) to rule out possible biases introduced by the random assignment of non-uniquely mapping reads (Supplementary Figure S5). These results suggest that LINE-1s are associated to chromatin opening and/or to the initiation of transcription at ZGA onset. To discriminate between these two contributions, we investigated the localisation of the ATAC peaks on the 3,731 LINE-1s overlapping early 2-cell intergenic open chromatin domains. Our results showed that the majority of these LINE-1s overlapped open chromatin domains at the 3’ and not at the 5’ end (Figure 3D). In addition, these LINE-1s appeared to be marked by open chromatin domains exclusively in the early 2-cell stage (Figure 3D) and no evidence of open chromatin was observed either upstream or downstream the LINE-1s of interest, confirming the specificity of the localisation of these open chromatin domains (Figure 3D). The enrichment of open chromatin at the 3’ of transcriptional units is of difficult interpretation without additional specific experiments. However, previous evidence suggested that open chromatin at transcription termination sites (TTSs) in early embryos denotes active transcription (Wu et al., 2016) as it reflects the binding and pausing of factors engaged in transcription termination, including RNA polymerase II (Pol-II) (Glover-Cutter et al., 2008). In line with this observation, no specific TFs were predicted to bind the open chromatin domains overlapping these LINE-1s (see Methods).

### Pol-II positioning suggests transcription of LINE-1 elements overlapping intergenic open chromatin domains in the early 2-cell stage might initiate at ZGA minor wave onset

**FIGURE 4:**
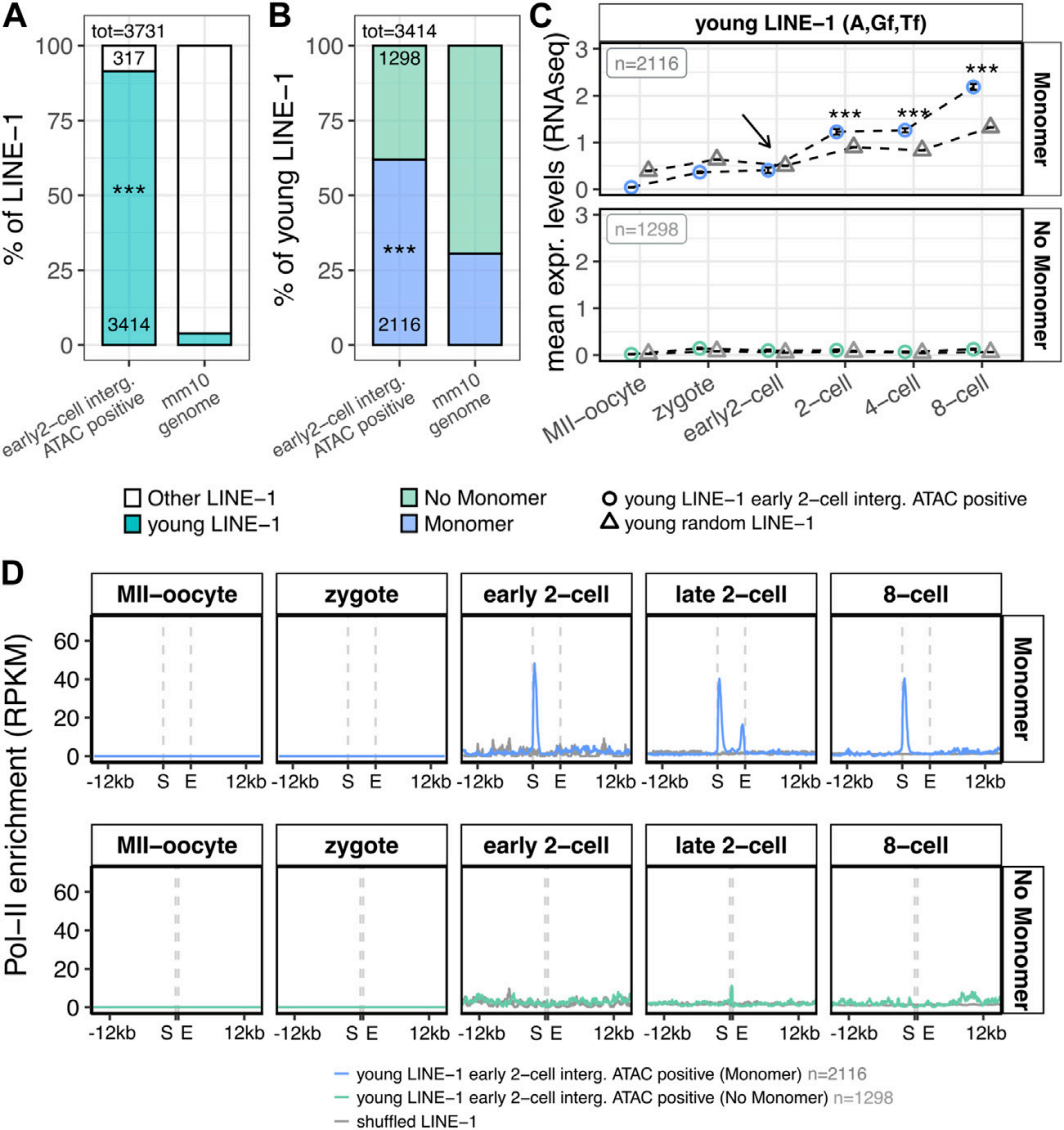
Structural, transcriptional and epigenetic characterisation of LINE-1 elements overlapping early 2-cell intergenic ATAC-seq peaks. **(A)** Subfamily classification of the 3,731 LINE-1s of interest. Left bar represents the percentage of the 3,731 LINE-1s identified in Figure 3C (overlapping intergenic early 2-cell ATAC-seq peaks) belonging to young LINE-1 subfamilies (A, G_*f*_, T_*f*_, blue) or to other LINE-1 subfamilies (white). Bar on the right represents the same, but for all the LINE-1s annotated in the murine reference genome. Numbers reported within the bars indicate the number of LINE-1s counted for each condition. Numbers above the bars indicate the total number of LINE-1s considered. (***p < 0.001, two-proportions z-test) **(B)** Presence/absence of monomers in the 3,414 young LINE-1s of interest. Left bar represents the percentage of the 3,414 young LINE-1s of interest (overlapping early 2-cell intergenic ATAC-seq peaks and belonging to young subfamilies) containing or not containing a A, G_*f*_ or T_*f*_, monomer (blue and light green, respectively). The bar on the right represents the same, but for all the young LINE-1s annotated in the murine reference genome. Numbers reported within the bars indicate the number of LINE-1s counted for each condition. Numbers above the bars indicate the total number of LINE-1s considered. (***p < 0.001, two-proportions z-test) **(C)** Transcriptional characterisation of the 3,414 young LINE-1s of interest (overlapping early 2-cell intergenic ATAC-seq peaks and belonging to young subfamilies). Normalised expression levels (TMM) of the young LINE-1s overlapping early 2-cell intergenic ATAC-seq peaks are reported for each analysed stage for the 2,116 LINE-1 elements with a monomer (upper panel, blue circles) and for the 1,298 LINE-1s without a monomer (bottom panel, light green circles) monomer. The same is depicted for randomly selected young LINE-1 elements (grey triangles). Expression levels are reported as mean between all the analysed LINE-1s and between the biological replicates of each stage. Black arrow indicates the early 2-cell stage, where chromatin opening is observed in correspondence of the LINE-1s of interest 3’ end, but no significant increased expression is observed compared to randomly selected young LINE-1s. (***p < 0.001, z-statistics) **(D)** Metagene plot showing the Pol-II enrichment on the genomic loci of the 3,414 young LINE-1s of interest. Pol-II enrichment is calculated on: i) young LINE-1s overlapping early 2-cell intergenic ATAC-seq peaks with a monomer (upper panel, blue, n = 2,116), ii) young LINE-1s overlapping early 2-cell intergenic ATAC-seq peaks without a monomer (bottom panel, light green, n = 1,298) and iii) LINE-1 coordinates shuffled for 1,000 times (grey). Enrichment of Pol-II is reported as RPKM (number of reads per bin/number of million mapped reads * bin length in kb). For the shuffle LINE-1s, mean signal among the randomisations is reported. S = start coordinate, E = end coordinate.

The murine genome is composed by evolutionary old (V, N, Mus and Lx, evolved 4–10 MY ago) and young (A, G_*f*_ or T_*f*_, 0.2–4 MY) LINE-1 elements and, while the former are usually represented by transcriptionally inactive elements, the latter are more likely to have retained their transcriptional competence (Sookdeo et al., 2013). Based on this evidence, we classified the previously identified LINE-1 elements overlapping early 2-cell intergenic ATAC-peaks according to the subfamily they belong to, which is a direct indicator of their evolutionary age. The results showed that 92% of such LINE-1s (3,414/3,731) belonged to evolutionary young subfamilies (A, G_*f*_ or T_*f*_), while these subfamilies represent only 4% of the total LINE-1s annotated in the murine genome (Figure 4A; Supplementary Table S7, *p* < 2.2e-16). Since most of the LINE-1 elements in the murine genome are 5’ truncated (Mouse Genome Sequencing Consortium et al., 2002), these young LINE-1s could still lack an intact promoter thus representing transcriptionally inactive fragments. Therefore, the 3,414 evolutionary young LINE-1s of interest were classified based on the presence or absence of intact A, G_*f*_ or T_*f*_ monomers in their internal promoters. To this end, the coordinates of the A, G_*f*_ or T_*f*_ monomers in the reference murine genome were retrieved from a previous publication (Zhou and Smith, 2019) and overlapped with the genomic coordinates of the LINE-1s of interest. Our results showed that 62% of the evolutionary young LINE-1s overlapping intergenic open chromatin domains at ZGA onset (2,116/3,414) contained an intact A, G_*f*_ or T_*f*_ monomer, whereas the same feature was displayed by only 30% of the young LINE-1 elements in the reference genome (Figure 4B; Supplementary Table S7, *p* < 2.2e-16). These results support the idea that the LINE-1s overlapping early 2-cell intergenic ATAC-peaks could be autonomously transcribed. To define the transcriptional dynamics of these LINE-1s, we interrogated again the RNA-seq data. To this end, we compared the expression levels of the 2,116 intact and 1,298 truncated LINE-1s overlapping early 2-cell intergenic ATAC peaks with the ones of randomly selected young LINE-1s. As expected, when lacking an A, G_*f*_ or T_*f*_ monomer in their internal promoters, both groups of LINE-1s resulted not to be expressed (Figure 4C, bottom).When containing A, G_*f*_ or T_*f*_ monomers, the 2,116 LINE-1s overlapping early 2-cell intergenic ATAC-seq peaks did not result significantly more expressed in the early 2-cell stage than randomly selected young LINE-1s (Figure 4C, top). Moreover, the expression levels of these 2,116 LINE-1s in the early 2-cell stage were low (<1 normalised read), resembling more transcriptional noise rather than a real expression and thus questioning whether these elements, despite showing chromatin accessibility at the early 2-cel stage, are really transcribed in the early 2-cell stage (Figure 4C, top). On the contrary, these LINE-1 RNAs, although lacking chromatin accessibility at these stages (Supplementary Figure S6), were significantly more expressed in the 2-, 4- and 8-cell stages, when compared to randomly selected young LINE-1s (Figure 4C, top). These results highlighted an apparent inconsistency between ATAC-seq and RNA-seq results. To solve this issue, we retrieved publicly available small-scale Tn5-assisted chromatin cleavage with sequencing (Stacc–seq) data to profile the Pol-II binding sites in the very same developmental stages (Liu et al., 2020). First, the genome-wide Pol-II binding sites in the entire dataset (from MII-Oocyte to 8-cell stage) were identified (Supplementary Table S8). Then, Pol-II binding profile nearby the genomic *loci* of the 2,116 LINE-1s of interest was analysed. Coherently with the expectations, Pol-II did not bind the LINE-1s of interest at any stage when they lacked monomers in their internal promoters (Figure 4D, bottom panel). On the contrary, Pol-II appeared to be enriched as early as in the early 2-cell stage on the young LINE-1s of interest when they had a monomer in their internal promoter region (Figure 4D upper panel; Supplementary Figure S7A, *p* < 2.2e-16). In addition, Pol-II was enriched also in the monomers of the LINE-1s of interest at the 2-cell stage (Figure 4D; Supplementary Figure S7A, *p* < 2.2e-16). Of note, at the 2-cell stage, Pol-II was enriched also at the 3’ end of such LINE-1s (Figure 4D; Supplementary Figure S7B, *p* < 2.2e-16). Since Pol-II positioning at the transcript 3’ end marks transcription termination (Glover-Cutter et al., 2008), it is likely that the transcription of such LINE-1s is terminated at the end of the late 2-cell stage, thus suggesting that the transcription of the LINE-1s of interest was initiated at the early 2- and terminated at the late 2-cell stage. However, Pol-II resulted unexpectedly enriched in the monomers of the 2,116 LINE-1s of interest also at the 8-cell stage (Figure 4D; Supplementary Figure S7A, *p* < 2.2e-16). Analysis of the specific set of LINE-1 monomers bound by Pol-II at the 8-cell stage revealed how 87% of such monomers were bound by Pol-II already at the early or late 2-cell stage (Supplementary Figure S7C). According to this data, the set of LINE-1s of interest bound by Pol-II at the 8-cell stage is roughly the same which is bound by Pol-II in the early and late 2-cell stages, suggesting the stalling of the Pol-II on these loci at the 8-cell stage, rather than their transcription. This is coherent with previous studies showing LINE-1 transcription exclusively at the 2-cell stage (Jachowicz et al., 2017; Percharde et al., 2018), and it is further corroborated by the observation that these LINE-1s showed chromatin accessibility exclusively at the early 2-cell stage (Figure 3D) and that a possible evidence of their transcription termination (Pol-II enrichment at the 3’ end) is observed exclusively at the late 2-cell stage, and not at the 8-cell stage (Figure 4D; Supplementary Figure S7B).

### YY1 is a potential candidate as transcriptional regulator at ZGA minor wave of young LINE-1s containing a monomer and mapping within intergenic open chromatin

**FIGURE 5:**
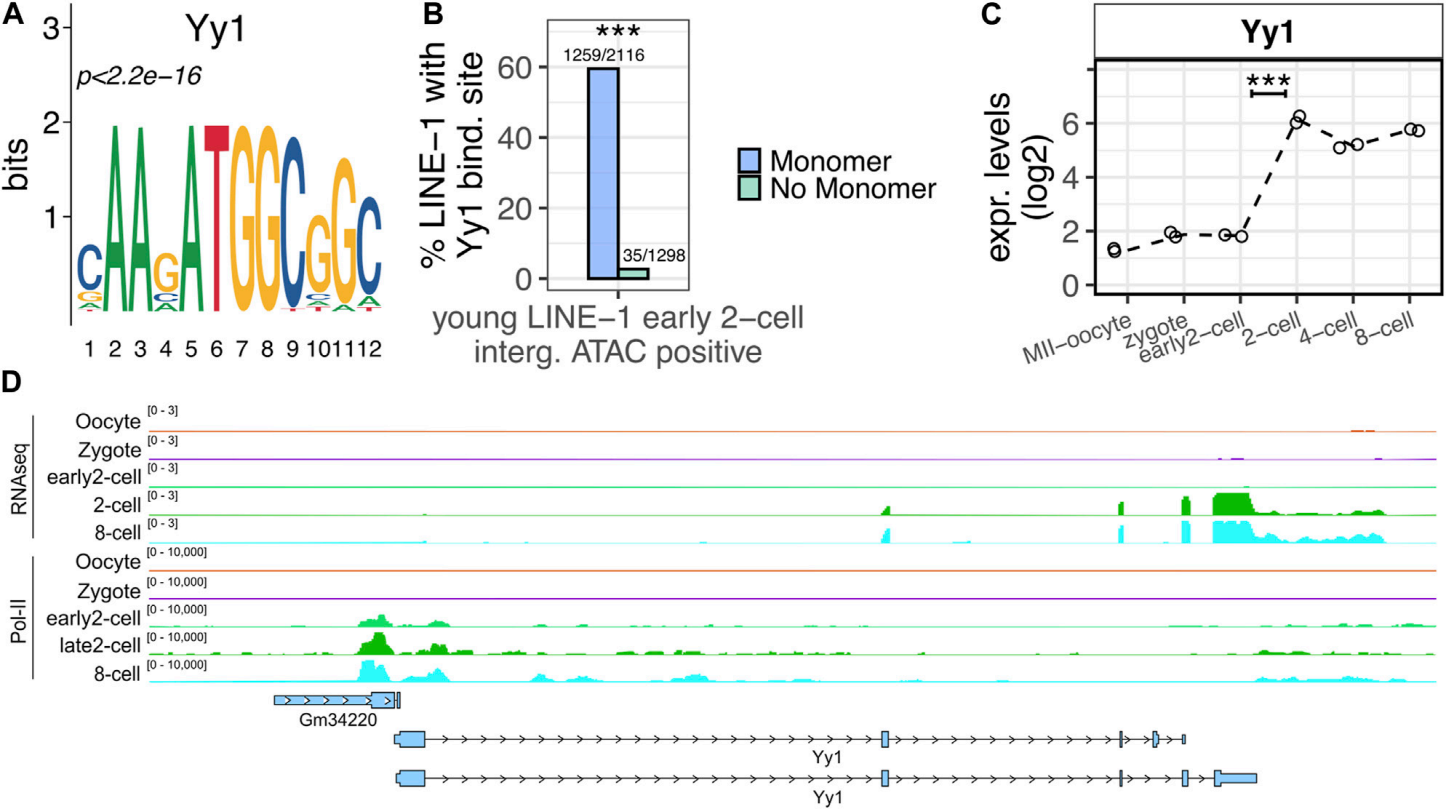
YY1 is predicted to bind LINE-1s of interest. **(A)** YY1 motif. YY1 is the known motif most significantly enriched in the monomer regions of the young LINE-1s containing a monomer and overlapping early 2-cell intergenic ATAC-seq peaks. **(B)** YY1 enrichment in LINE-1s of interest. Left bar (blue) reports the percentage of the 2,116 young LINE-1s containing a monomer and overlapping early 2-cell intergenic ATAC-seq peaks predicted to bind YY1 in their monomer region is reported on y-axis. Right bar (light green) reports the same, but for the 1,298 young LINE-1s overlapping early 2-cell intergenic ATAC-seq peaks and not containing a monomer. (***p < 0.001, two-proportions z-test). **(C)** *Yy1* expression levels. Normalised expression levels (TMM) of *Yy1* are reported on y-axis for all the analysed stages. Data points indicate the *Yy1* expression in the two replicates analysed for each stage. Dashed line connects the mean expression levels calculated between the two replicates of each stage. Expression levels are reported as log_2_(TMM+1). (***p < 0.001 and log_2_FC > 1). **(D)** Screenshot showing *Yy1* RNA-seq and Pol-II binding sites normalised signal. The first five tracks report the normalised expression levels from RNA-seq data. The last five, the Pol-II binding profile. Annotated genes and TEs (blue) are also reported.

Since our ATAC-seq and Pol-II binding sites analysis suggested that the young LINE-1s overlapping early 2-cell open chromatin domains and containing an A, G_*f*_ or T_*f*_ monomer were actively transcribed at ZGA onset, we wondered which TF might be responsible for their transcriptional activation. To this end, a motif enrichment analysis was performed searching for known motifs predicted to bind the monomer sequences of the 2,116 young LINE-1s containing a monomer and overlapping early 2-cell open chromatin domains, using as background the sequences of the 1,298 young LINE-1s overlapping open chromatin domains in the early 2-cell stage but not containing a monomer in their promoter sequence. According to this analysis, the most significantly enriched motif in the monomer containing group was YY1, a well-known TF binding LINE-1 monomers (Figure 5A, *p* < 2.2e-16) (Athanikar et al., 2004). In particular, while 60% (1,259/2,116) of the LINE-1s of interest carrying a monomer were predicted to contain YY1 binding site, this was observed for only 3% (35/1,298) of the LINE-1s with the same features as the ones of interest but lacking a monomer in their promoter (Figure 5B, *p* < 2.2e-16). We next sought to define the transcriptional profile of *Yy1* in the preimplantation embryo. According to the analyzed RNA-seq data, *Yy1* mRNA appeared to be part of the maternal transcripts deposited in the oocyte cytoplasm, as its mRNA is already detectable, although at very low levels, in the transcriptionally inactive MII-Oocyte and zygote stages (Figure 5C). In addition, RNA-seq data showed that *Yy1* expression was significantly increased between the early 2- and the 2-cell stage, suggesting that *Yy1* transcription is activated upon ZGA major wave (Figure 5C). However, Pol-II binding data indicated that Pol-II bound *Yy1* TSS as early as in the early 2-cell stage (Figure 5D).

## Discussion

The role of MERVL elements as potent regulators of murine ZGA is well known (Peaston et al., 2004; Macfarlan et al., 2012). However, another class of TEs, LINE-1 elements, recently came under the spotlight as key regulator of murine ZGA (Jachowicz et al., 2017; Percharde et al., 2018). Yet, how LINE-1 transcription is activated and how, and if, the transcriptional program of these elements differs from the one of MERVL is unclear. To confront these important molecular questions, we integrated RNA-seq, ATAC-seq and Stacc–seq data to characterise MERVL and LINE-1 transcriptional dynamics during murine ZGA at the transcriptional and epigenetic levels.

The transcriptional characterisation of the murine ZGA highlights a remarkable number of both genes (861) and TEs (2,589) transcriptionally activated at ZGA minor wave. On the one hand, our results confirm previous evidence highlighting that ZGA minor wave genes are enriched in MERVL elements nearby their TSS (Macfarlan et al., 2012) and that MERVL undergo transcriptional activation at ZGA onset (De Iaco et al., 2017; Hendrickson et al., 2017). On the other hand, our analysis highlights new genomic features characterising the ZGA minor wave genes. In particular, the 861 ZGA minor wave genes result enriched in gene-dense compartments such as gene clusters. Although the function of gene clusters is still unclear, we speculate that, likewise MERVL, gene clusters could act as single regulators controlling multiple genes at the same time. In addition, the colocalization of different copies of similar and co-expressed genes might ensure the production of the needed molecular effector even in cases of mutations inactivating one of the members of the cluster. Moreover, since gene clusters are transcriptionally dense genomic compartments where multiple activators are bound in a tight genomic portion, it could also be proposed that such a high density of activating factors facilitates the initiation of the embryo transcription, as recently observed in zebrafish (Hadzhiev et al., 2023).

Our data, together with previous evidence (Peaston et al., 2004; Malki et al., 2014), show that young LINE-1 transcripts are already present in the embryo cytoplasm ahead of fertilisation, as LINE-1s belong to the set of maternal transcripts deposited in the oocyte cytoplasm, making it difficult to detect upregulated young LINE-1s in early 2-cell compared to zygote stages to identify the elements first transcribed by the embryo. To investigate the possible zygotic transcription upon ZGA minor wave of LINE-1s, we integrated RNA-seq data with ATAC-seq data of the same developmental stages. The analysis of the ATAC-seq data reveals that, specifically at the ZGA onset (early 2-cell), the identified chromatin domains are enriched in intergenic regions (50% of the total peaks). Of the intergenic open chromatin domains identified at ZGA onset, 50% specifically overlapped LINE-1 elements, with no other stages displaying a similar feature. These results indicate that more than 25% of the total open chromatin domains identified at ZGA onset are located in intergenic regions in correspondence of LINE-1s. The investigation of the localisation of these open chromatin domains with respect to LINE-1 *loci*, reveals that the ATAC-seq peaks are preferentially located at the 3’ end of the LINE-1s of interest. Although the interpretation of open chromatin at the 3’ end of genes/TEs still remains elusive, previous evidence suggests that this phenomenon, at least in early embryos, denotes active transcription (Wu et al., 2016) as it reflects the binding and pausing of factors engaged in transcription termination, including RNA polymerase II (Pol-II) (Glover-Cutter et al., 2008). In addition, LINE-1s within intergenic open chromatin domains result enriched in evolutionary young elements containing an intact monomer in their internal promoter, a pre-requisite for active transcription. These results support the idea that LINE-1s are transcribed at ZGA onset. However, when interrogating again the RNA-seq dataset comparing the expression levels of these LINE-1s with a set of randomly selected elements, no significant differences were observed at the early 2-cell stage, but only in the 2- 4- and 8-cell stages, although chromatin accessibility on these LINE-1s is observed specifically at the early 2-cell stage.

This lack of consistency between RNA-seq and ATAC-seq data can be explained in a model in which the protein of a transcriptional activator for these LINE-1s, such as YY1, is not present at the early 2-cell stage. Interestingly, proteomic data (Israel et al., 2019) show that YY1 expression reaches its top of expression at the 2-cell stage (Israel et al., 2019) supporting the proposed model. This model, however, does not explain how at the 2-, 4- and 8-cell stages the transcription of the LINE-1s of interest occurs despite the lack of accessible chromatin. Here methodological differences can be taken into consideration. In the analysed RNA-seq library, the sequenced transcripts were selected based on the presence of the poly-A tail, thus the sequenced library was composed exclusively of mature transcripts. LINE-1 transcripts detected at 2-, 4- and 8-cell stage should not be considered necessarily transcribed in the very same stage as their transcription could have begun in earlier stages (*i.e.*, early 2-cell) while the maturation happened in later ones. We therefore believe that, at least in this highly dynamic time-course dataset, it is difficult to make direct correlations between ATAC-seq and RNA-seq dataset as the former is probably measuring chromatin accessibility (transcription occurrence/priming) whereas the latter is possibly detecting mature transcripts (transcription termination). Although reasonable, our model is speculative and based exclusively on meta-analysis and therefore needs to be properly validated.

To further investigate the transcriptional dynamics of the intergenic LINE-1s containing monomers localized within open chromatin at ZGA, we retrieved Pol-II Stacc-seq data identifying Pol-II binding during mouse preimplantation development. Remarkably, Pol-II is significantly enriched in the monomers of the LINE-1s of interest with respect to the rest of the genome as early as at ZGA onset, whereas it does not bind LINE-1s lacking a monomer in their internal promoters. In addition, Pol-II is enriched at both 5’ and 3’ ends of the LINE-1s of interest at the 2-cell stage and at the 5’ end of these LINE-1s at the 8-cell stage. Since Pol-II enrichment at the TTS marks transcriptional termination and that the LINE-1s elements bound by Pol-II at the 8-cell stage were the same bound at early 2- or late 2-cell stage, we hypothesise that LINE-1 transcription started at the early 2-cell stage and terminated at the late 2-cell stage with Pol-II stalling on the monomers of a portion of these LINE-1s, without transcribing them, at the 8-cell stage.

Finally, our motif enrichment analyses predicts that YY1, a well-known TF binding LINE-1 monomers (Athanikar et al., 2004), could be the TF activating the transcription of these LINE-1s. Although this observation only results from an *in silico* prediction, it is interesting to observe how YY1 was previously described to contribute to the silencing of ERVs in mouse embryonic stem cells (Guallar et al., 2012; Schlesinger et al., 2013). Thus, it is possible that YY1 might participate to the repression of the *Dux*/MERVL transcriptional program by acting through two pathways. On the one hand, YY1 indirectly switches-off the *Dux*/MERVL pathway by activating the transcription of LINE-1s, whose RNAs participate to *Dux*/MERVL pathway silencing (Percharde et al., 2018). On the other one, YY1 directly participates to *Dux*/MERVL silencing as described in mouse embryonic stem cells (Guallar et al., 2012; Schlesinger et al., 2013) reinforcing the LINE-1 silencing activity. Again, it is worth mentioning that these results derive from a pure computational analysis thus in need of further experimental validation.

The major limitation of our computational study is given by the usage of short reads to infer signals from transposable elements. Using short reads to map the activity of TEs is difficult due to the repetitive nature of these elements. Being sure of the location of a read from a repetitive element is not always possible. There is no solution to this problem, especially considering that the use of uniquely mapping reads alone often prevents the identification of signals coming from certain portions of repeated elements, generally the youngest and most active ones. However, the lack of a solution to this problem should not prevent the execution of exploratory analyses and their usage to propose models to be validated with alternative and more specific techniques. Models that were unknown before and that could be biologically important to increase our knowledge in a given topic. In our study, the uncertainty about the specific position of a certain portion of reads was mitigated by the robustness of the identified enrichments that resulted to characterize the majority of the TEs belonging to a specific subgroup (young LINE-1s) in a specific developmental stage (early 2-cell). Even if we could not be sure about the exact location of each specific elements active at a given time, our analysis allowed us to understand that young LINE-1 elements show specific features as a group, which, according to us, paves the way for future and more focused analysis.

In summary, we have identified a set of evolutionary young LINE-1 elements ready to be transcribed at ZGA minor wave onset marked by chromatin accessibility at the 3’ ends and Pol II binding at the promoter regions. In particular, these LINE-1s are confined in intergenic regions, in contrast with *Dux*/MERVL targets whose transcription is started from gene-dense compartments. Altogether, our results suggest that *Dux*/MERVL and LINE-1 transcriptional pathways are two distinct and antagonistic pathways whose activation at ZGA minor wave is started from spatially separated genomic compartments of the murine genome.

## Data Availability

The original contributions presented in the study are included in the article/Supplementary Material, further inquiries can be directed to the corresponding authors. All the analysed-omic datasets were retrieved from previous publications: RNA-seq: https://www.ebi.ac.uk/ena/browser/view/PRJNA277361 ATAC-seq: https://www.ebi.ac.uk/ena/browser/view/PRJNA277362 Stacc-seq: https://www.ebi.ac.uk/ena/browser/view/PRJNA558961.

## Supplementary material

Supplementary Figure S1 TE enrichment in the TSS of the minor wave genes. TSS coordinates of the 861 early 2-cell / zygote up-regulated genes (minor wave genes) have been elongated by 100 nt at both ends and overlapped with the coordinates of all the TEs annotated in the mm10 genome. The number of overlaps for each TE subfamily is indicated by the dark-coloured bars (Observed). The same analysis was repeated on randomly selected genes (Expected, light-coloured bars, 1,000 randomisations, mean +/− sd is reported). Only enriched, and not depletion, are considered as significant (****p*<2.2e-16, z-statistics).

Supplementary Figure S2 Number of down- and up-regulated TEs in early 2-cell vs zygote. **(A)** Number of TE loci down (left panel) and up-regulated (right panel) in early 2-cell vs zygote for each TE subfamily. The number of DE loci is also reported on the top of each bar. “young LINE-1” refers to LINE-1 elements belonging to A, Gf or Tf subfamilies. “old LINE-1” refers to all the other LINE-1 elements. **(B)** Number of old LINE-1s up-regulated between early 2-cell and zygote stages. The number of up-regulated LINE-1 loci for each old LINE-1s subfamily is reported on y-axis. The old LINE-1s here represented are the same LINE-1s classified as “old LINE-1” in panel A (*n* - 136). **(C)** Distance of the 136 old LINE-1s from the closest gene. Histogram reporting the distance in nucleotides between each of the 136 up-regulated old LINE-1s and the closest annotated gene. Each bin groups 3 kb.

Supplementary Figure S3 TE consensus expression levels. Heatmap reporting the normalised expression levels for all the TE consensus annotated in the murine genome. TE consensus with no expression in any of the analysed stages were removed. Expression values are reported as TMM (edgeR’s normalised counts) in log2 scale. Pseudocount of 1 was summed to TMM to prevent infinite values after log transformation. Names of the TE consensus are reported on the right of the heatmap. TEs are grouped according to major TE subfamilies (Alu, IAP, MERVL, other ERV, young LINE-1 and all the other LINE-1 elements).

Supplementary Figure S4 Detection of young LINE-1 transcripts in transcriptionally blocked embryos. Normalised expression levels (TMM) of young LINE-1 element consensus sequences are reported for embryos treated with alpha-amanitin. Alpha-amanitin treatment was started at oocyte stage and RNA-seq sequencing performed at 1-cell (left panel) and late 2-cell stage (right panel). The different TE consensus sequences of each young LINE-1 subfamilies are reported on x-axis. Data points indicated the expression values for each biological replica (*n* = 4). In these embryos the endogenous transcription, and consequently ZGA, has been blocked. Therefore, the signal detected from LINE-1 transcripts derives exclusively from maternally supplied transcripts originally deposited into the oocyte cytoplasm.

Supplementary Figure S5 LINE-1 enrichment in intergenic regions at early 2-cell is confirmed by three independent analyses. **(A, B)** The same analysis performed to generate panels A and B of Figure 3 was repeated by performing an independent analysis by mapping the reads to the reference genome by using bwa. **(C, D)** Same as in A and B, but reads were mapped to the reference genome by bowtie2. **(E, F)** Same as in A and B, but reads were mapped to the reference genome by bowtie1. This was done in order to rule out possible biases introduced by the random assignment of non-uniquely mapping reads.

Supplementary Figure S6 Number of 2,116 LINE-1s of interest overlapping ATAC-seq peaks. The number of the 2,116 LINE-1s of interest (young LINE-1s with a monomer, overlapping early 2-cell intergenic ATAC-peaks) overlapping ATAC-seq peaks in each analysed stage are reported on x-axis.

Supplementary Figure S7 Enrichment of Pol-II in the 2,116 young LINE-1s with a monomer overlapping early 2-cell intergenic ATAC-seq peaks. **(A)** Pol-II enrichment in the monomers of the LINE-1s of interest. Blue bars represent the number of young LINE-1s early 2-cell intergenic ATAC-positive containing a monomer which overlap Pol-II binding sites (+/− 100 nt). Grey bars represent the same, but the overlap was performed between the coordinates of the monomer sequences of the LINE-1s of interest and shuffled Pol-II coordinates (1,000 times). In shuffled bars, mean +/− sem among the 1,000 randomisations is reported **(B)** Same as in **(A)**, but 3’ end coordinates (TTS +/− 100 nt), instead of monomer, were considered. **(C)** Upset plot representing the number of monomers of the 2,116 LINE-1s of interest that are bound by Pol-II in early 2-cell, late 2-cell and 8-cell stages. “Set size” indicates the total number of monomers of the LINE-1s of interest bound by Pol-II (the same as indicated by blue bars in panel A). Vertical bars indicate the number of monomers commonly or individually bound by Pol-II in each of the three stages of interest. (****p* < 0.001, z-statistics, in both A and B).

Supplementary Table S1 DE gene analysis results. Beside statistics from the edgeR analysis, the normalized expression levels (TMM) are reported. In addition, each gene was marked as Macfarlan positive or negative, ERVL positive or negative and the localization inside or outside a gene cluster is also reported.

Supplementary Table S2 TEs enriched nearby the TSS (+/−100 nt) of the 861 ZGA minor wave genes.

Supplementary Table S3 Genomic coordinates of the gene clusters as identified by ClusterScan.

Supplementary Table S4 DE results, TE loci analysis (SQuIRE).

Supplementary Table S5 DE results, TE consensus analysis (TEspeX).

Supplementary Table S6 Genomic coordinates of the significantly identified ATAC-seq peaks.

Supplementary Table S7 Genomic coordinates of the 3,731 LINE-1s overlapping intergenic early 2-cell ATAC-seq peaks.

Supplementary Table S8 Genomic coordinates of the significantly identified Stacc-seq Pol-II binding sites.
