# Supplementary figures and images for: In silico characterisation of minor wave genes and LINE-1s transcriptional dynamics at murine zygotic genome activation
Source: Front Cell Dev Biol. 2023 Jun 14;11:1124266. doi: 10.3389/fcell.2023.1124266 (PMC10300423; doi:10.3389/fcell.2023.1124266)

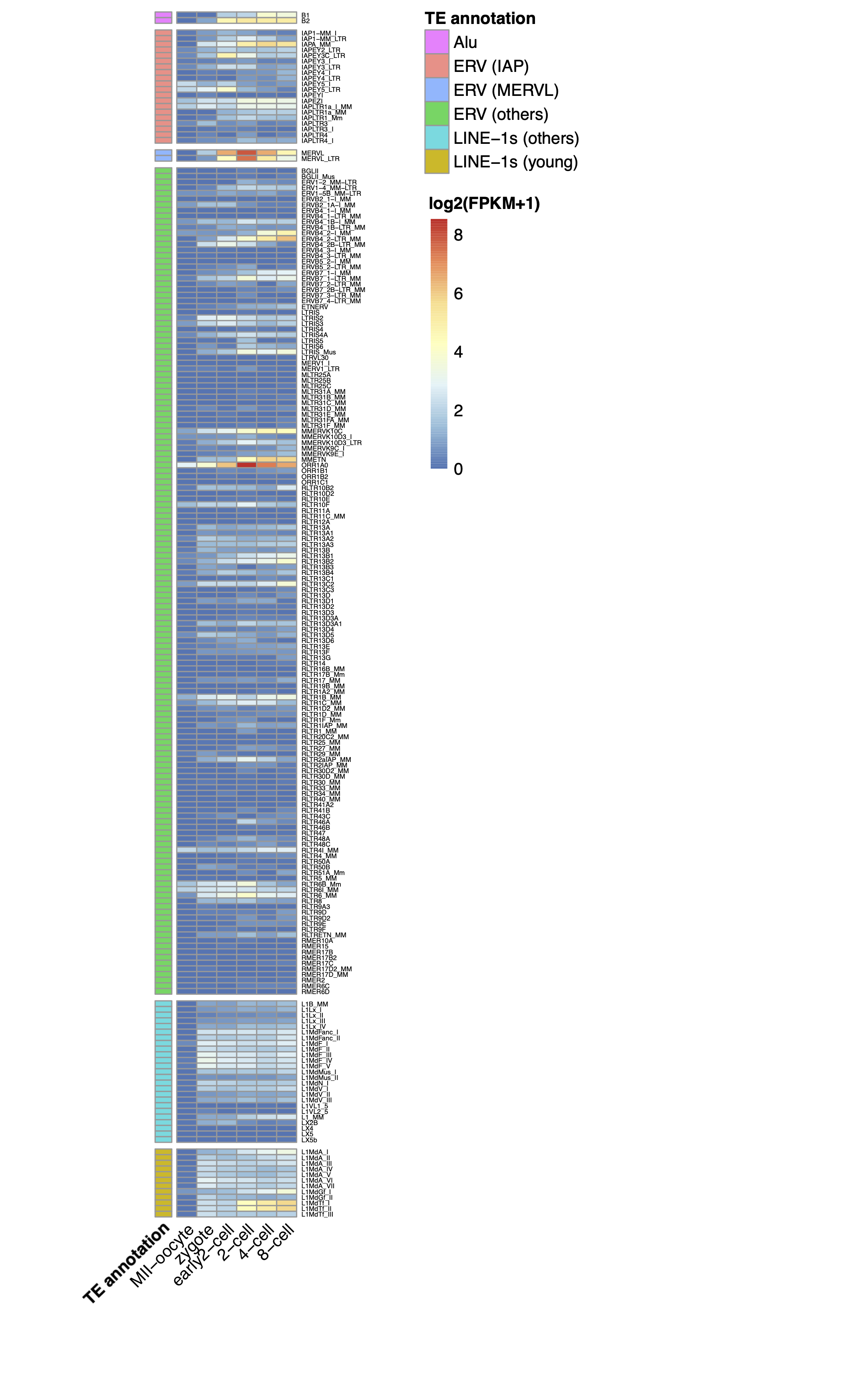

Supplement: Supplementary file 1 [file Image3.TIFF]

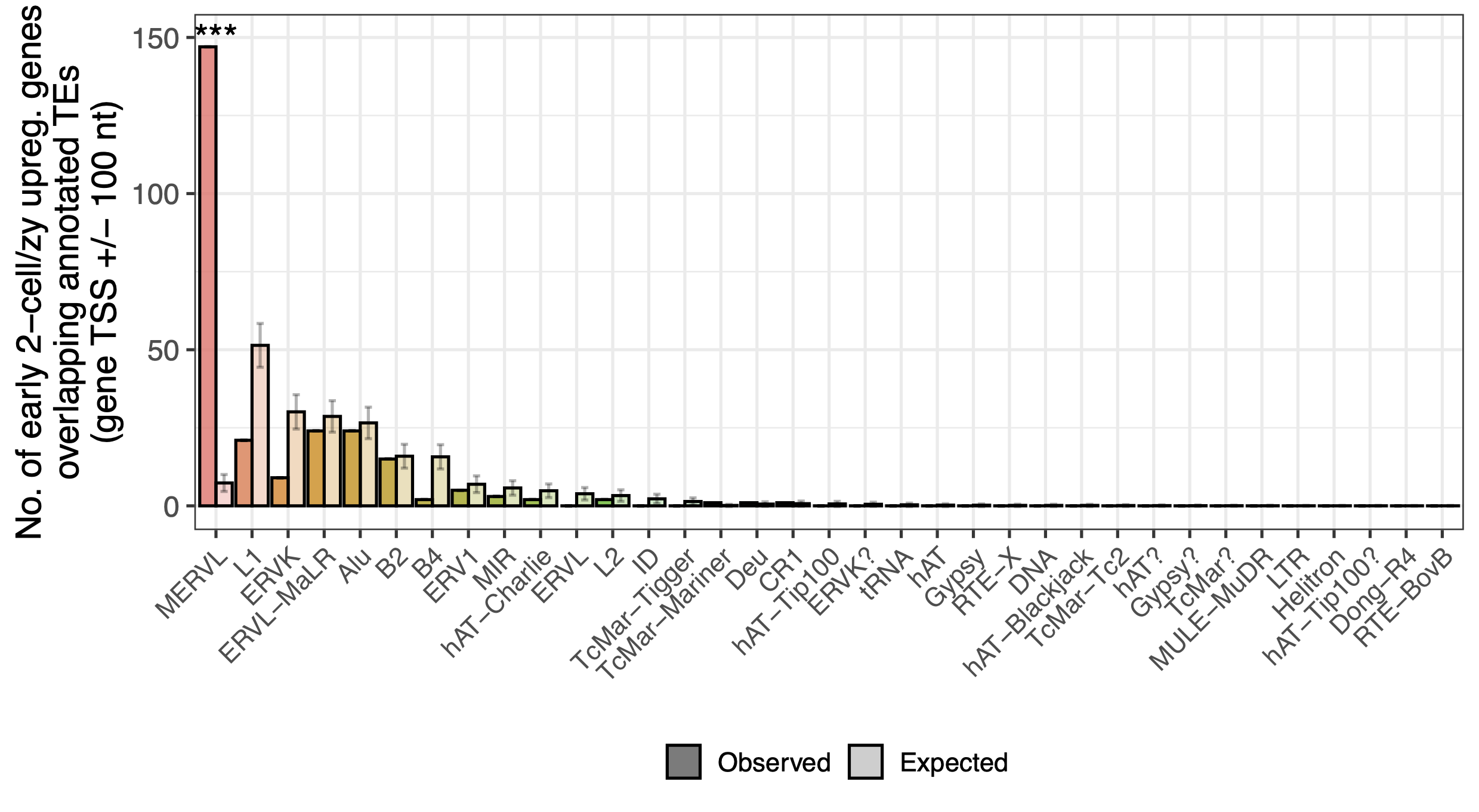

Supplement: Supplementary file 2 [file Image1.TIFF]

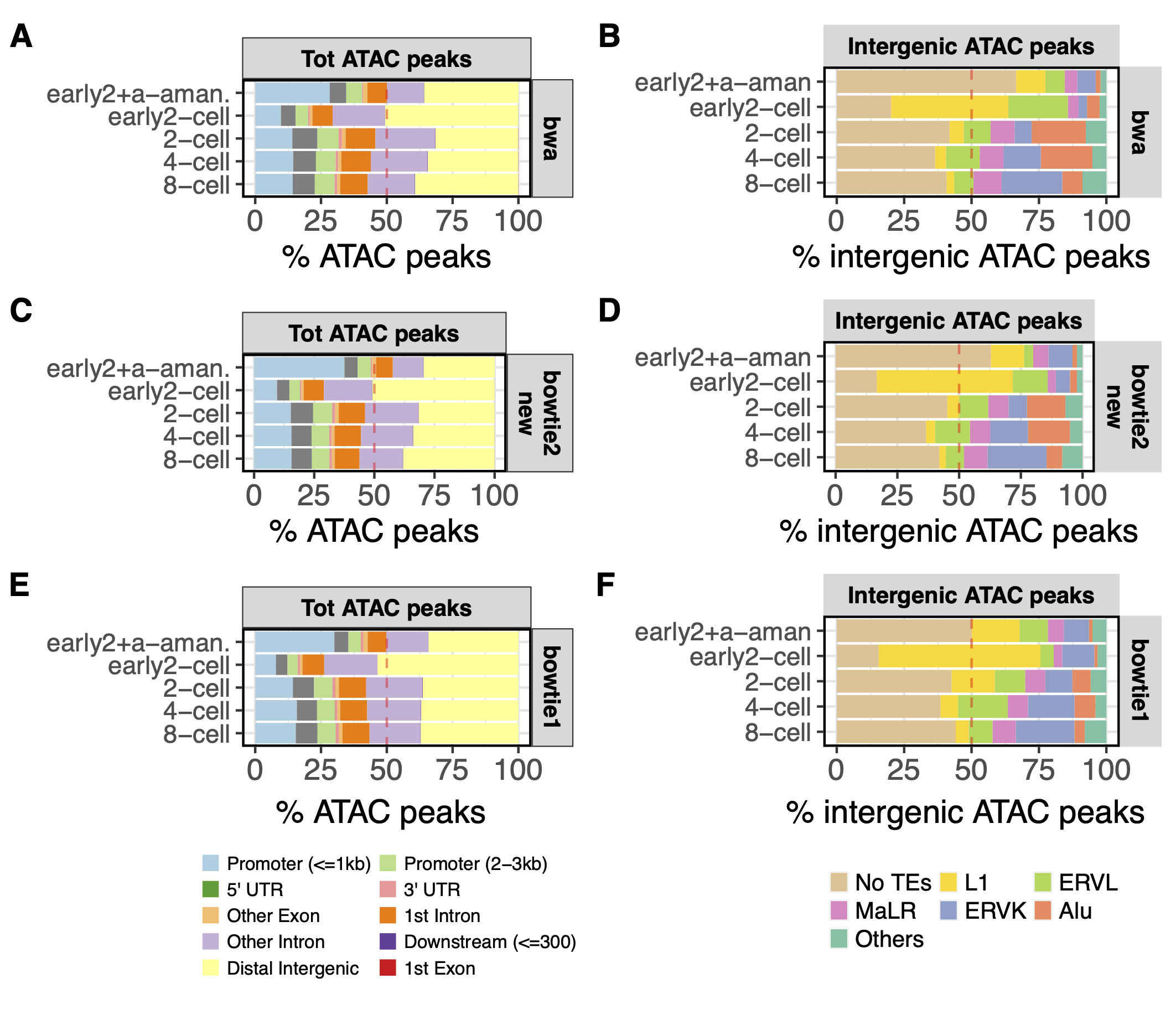

Supplement: Supplementary file 6 [file Image5.TIFF]

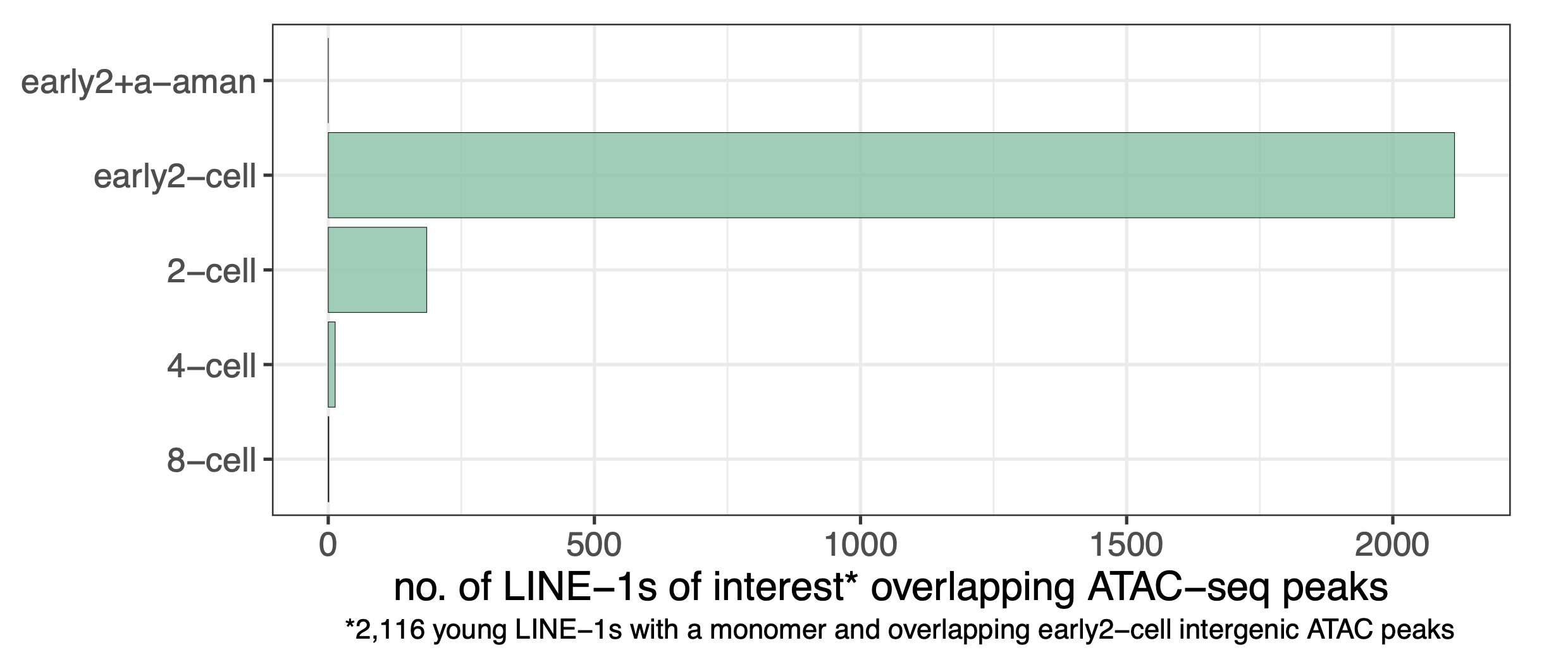

Supplement: Supplementary file 9 [file Image6.TIFF]

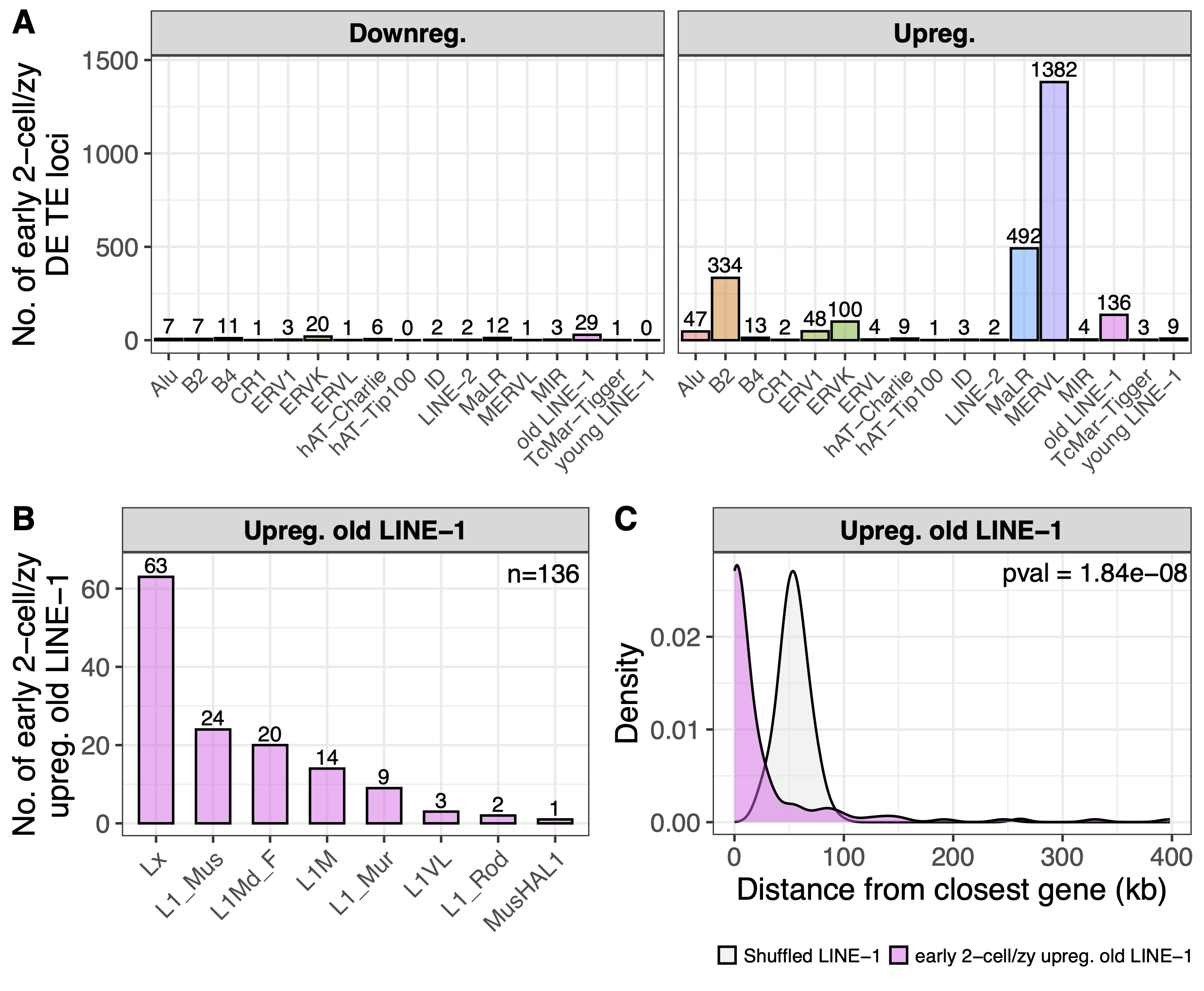

Supplement: Supplementary file 11 [file Image2.TIFF]

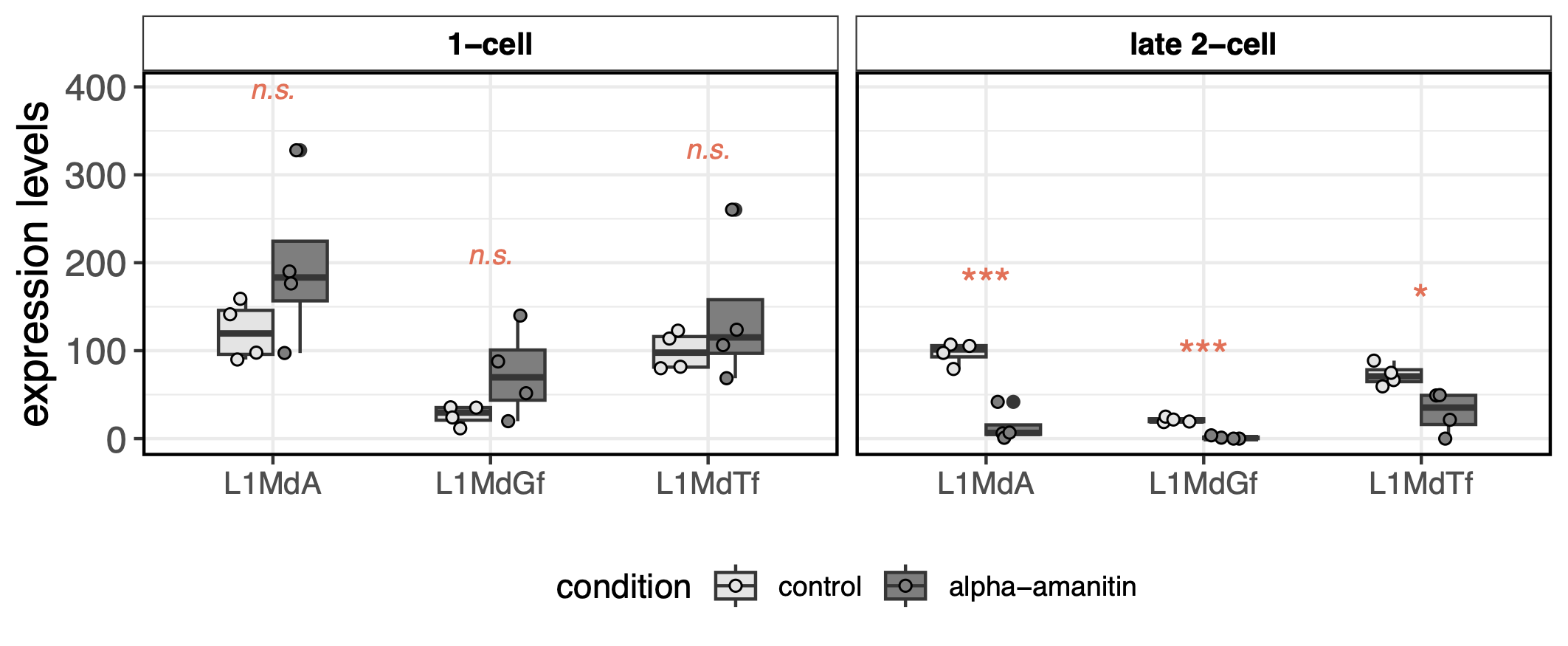

Supplement: Supplementary file 12 [file Image4.TIFF]

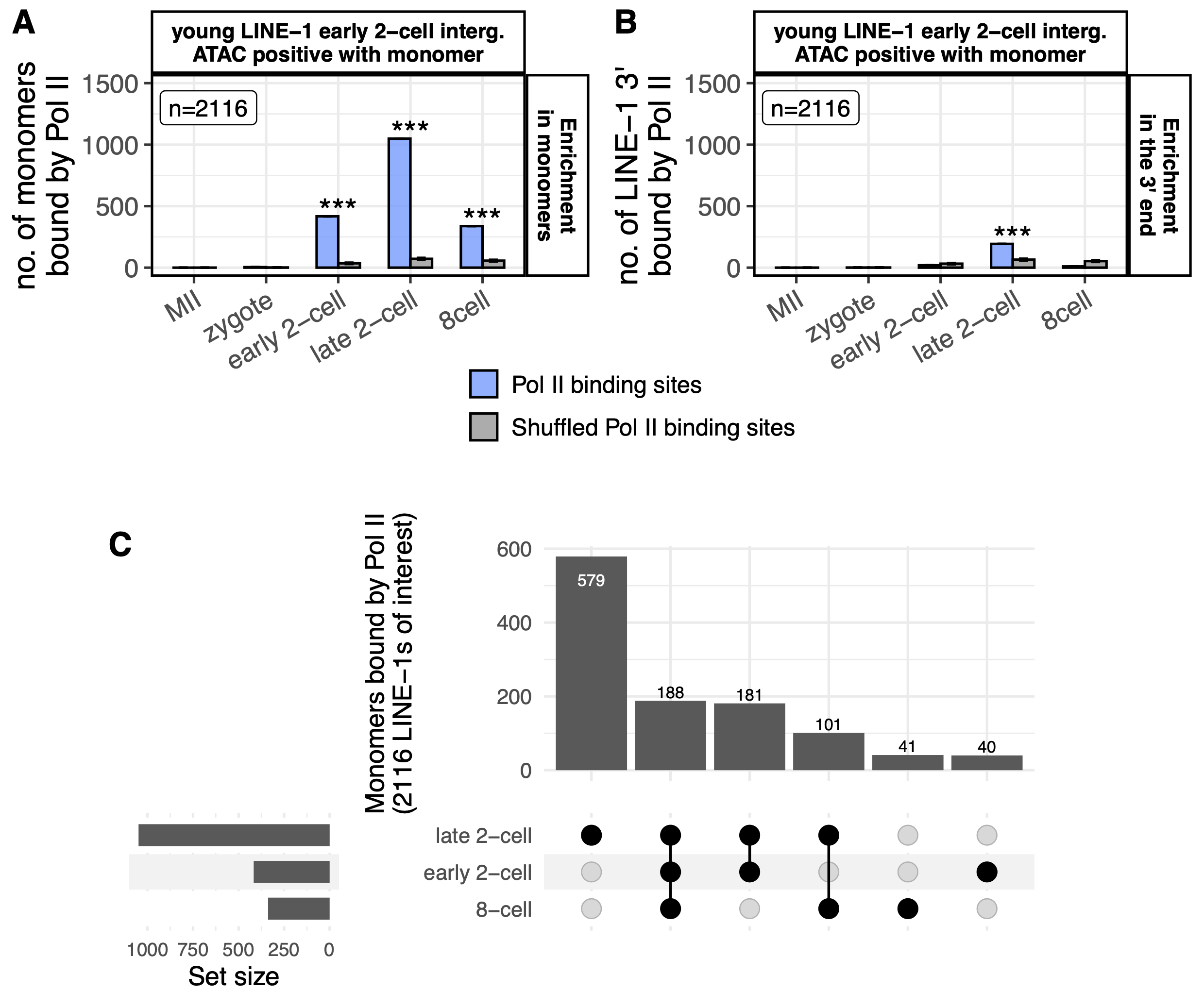

Supplement: Supplementary file 14 [file Image7.TIFF]
